# A Pilot Integrative Analysis of Colonic Gene Expression, Gut Microbiota, and Immune Infiltration in Primary Sclerosing Cholangitis-Inflammatory Bowel Disease: Association of Disease With Bile Acid Pathways

**DOI:** 10.1093/ecco-jcc/jjaa021

**Published:** 2020-02-04

**Authors:** Mohammed Nabil Quraishi, Animesh Acharjee, Andrew D Beggs, Richard Horniblow, Chris Tselepis, Georgios Gkoutos, Subrata Ghosh, A E Rossiter, Nicholas Loman, Willem van Schaik, David Withers, Julian R F Walters, Gideon M Hirschfield, Tariq H Iqbal

**Affiliations:** 1 Institute of Cancer and Genomic Sciences, University of Birmingham, Birmingham, UK; 2 Department of Gastroenterology, Queen Elizabeth Hospital, University Hospitals Birmingham, Birmingham, UK; 3 University of Birmingham Microbiome Treatment Centre, University of Birmingham, Birmingham, UK; 4 Centre for Liver and Gastroenterology Research, NIHR Birmingham Biomedical Research Centre, University of Birmingham, Birmingham, UK; 5 Institute of Translational Medicine, University Hospitals Birmingham, Birmingham, UK; 6 MRC Health Data Research UK [HDR UK], Wellcome Trust, London, UK; 7 NIHR Experimental Cancer Medicine Centre, NIHR Surgical Reconstruction and Microbiology Research Centre, Birmingham, UK; 8 Institute of Microbiology and Infection, University of Birmingham, Birmingham, UK; 9 Institute of Immunology and Immunotherapy, University of Birmingham, Birmingham, UK; 10 Division of Digestive Diseases, Imperial College London, London, UK; 11 Toronto Centre for Liver Disease, University of Toronto, Toronto General Hospital, Toronto, ON, Canada

**Keywords:** Autoimmune liver disease, dysbiosis, bioinformatics, colitis

## Abstract

**Background:**

Although a majority of patients with PSC have colitis [PSC-IBD; primary sclerosing cholangitis-inflammatory bowel disease], this is phenotypically different from ulcerative colitis [UC]. We sought to define further the pathophysiological differences between PSC-IBD and UC, by applying a comparative and integrative approach to colonic gene expression, gut microbiota and immune infiltration data.

**Methods:**

Colonic biopsies were collected from patients with PSC-IBD [*n *= 10], UC [*n* = 10], and healthy controls [HC; *n *= 10]. Shotgun RNA-sequencing for differentially expressed colonic mucosal genes [DEGs], 16S rRNA analysis for microbial profiling, and immunophenotyping were performed followed by multi-omic integration.

**Results:**

The colonic transcriptome differed significantly between groups [p = 0.01]. Colonic transcriptomes from HC were different from both UC [1343 DEGs] and PSC-IBD [4312 DEGs]. Of these genes, only 939 had shared differential gene expression in both UC and PSC-IBD compared with HC. Imputed pathways were predominantly associated with upregulation of immune response and microbial defense in both disease cohorts compared with HC. There were 1692 DEGs between PSC-IBD and UC. Bile acid signalling pathways were upregulated in PSC-IBD compared with UC [*p* = 0.02]. Microbiota profiles were different between the three groups [*p* = 0.01]; with inferred function in PSC-IBD also being consistent with dysregulation of bile acid metabolism. Th17 cells and IL17-producing CD4 cells were increased in both PSC-IBD and UC when compared with HC [*p* < 0.05]. Multi-omic integration revealed networks involved in bile acid homeostasis and cancer regulation in PSC-IBD.

**Conclusions:**

Colonic transcriptomic and microbiota analysis in PSC-IBD point toward dysregulation of colonic bile acid homeostasis compared with UC. This highlights important mechanisms and suggests the possibility of novel approaches in treating PSC-IBD.

## 1. Introduction

Primary sclerosing cholangitis [PSC] is characterised by progressive inflammation and fibrotic stricturing of the biliary tree.^1^ The pathogenesis of PSC is poorly understood, with no medical treatments, but is presumed to result from the complex interplay of immune dysregulation, gut microbial dysbiosis, and changes in bile acid homeostasis, in genetically predisposed individuals.^2–4^ PSC is highly comorbid with inflammatory bowel disease [IBD], which is ultimately diagnosed in approximately 75% of patients, primarily resembling ulcerative colitis [UC]. PSC-IBD is phenotypically different from UC, being more likely a pancolitis [especially right-sided] that follows a milder disease course but is associated with a significantly higher risk of colorectal cancer.

Genome-wide association studies [GWAS] have identified multiple shared and non-shared genetic loci that underlie the risk of developing PSC-IBD.^5–7^ A substantial component of the genetic architecture of PSC-IBD is not shared with UC, and genetic correlation modelling generates a UC comorbidity rate of only 1.6% in patients with PSC.^5^ Furthermore, patients with PSC-IBD have distinct gut microbial profiles compared with UC.^2–4^ These differences have led to the proposal that colonic inflammation in PSC-IBD and in UC results from different pathways. The colonic mucosal gene expression profile [transcriptome] in PSC-IBD, has not previously been explored.

To appreciate the complex and interdependent mechanisms underlying the colonic presentation of PSC-IBD, we applied a comparative systems biology approach, capturing the colonic mucosal transcriptome, mucosally adherent gut microbiota profiles, and mucosal immunophenotype in patients with PSC-IBD, UC alone, and healthy controls [HC] in this pilot study. Through this approach, we modelled interactions between different biological systems to interrogate key processes driving the phenotypes observed in PSC-IBD and UC.

## 2. Methods

### 2.1. Study population and sample collection

Patients with PSC-IBD, with UC, and HC were recruited from clinic and endoscopy lists. PSC-IBD and UC were documented in keeping with European guidelines on diagnosis [EASL and ECCO]. Healthy subjects had no known comorbidities and normal colonoscopy [other than haemorrhoids] as part of investigation for rectal bleeding. Subjects were excluded if they had taken antibiotics and/or probiotics in the past 3 months. Only patients with large-duct PSC were recruited as part of the PSC-IBD cohort, and alternative aetiologies were excluded for all patients. Recurrent PSC was documented in transplant patients, and secondary causes were excluded by standard clinical workup. Colonic mucosal biopsies were taken from the sigmoid colon and collected on ice, in both Qiagen RNAlater tubes [for storage in -80 C] and Miltenyi Biotec gentleMACS C-Tubes containing complete RPMI media [for immediate processing]. Ethical approval was given by University of Birmingham Human Biomaterials Resource Centre [HTA Licence 12,358]. An overview of the methodology for this study is summarised in Supplementary Figure 1, available as Supplementary data at *ECCO-JCC* online.

### 2.2. RNA library preparation and sequencing

DNA and RNA were extracted from mucosal biopsies within 2 weeks of collection. A modified protocol of Qiagen AllPrep DNA/RNA Mini Kit that included mechanical lysis and on-column DNAse digestion was used [detailed methodology in Supplementary Methods, available as Supplementary data at *ECCO-JCC* online]. Ribo-Zero Gold rRNA Removal Kit [Illumina, San Diego, USA] was used to remove contaminating ribosomal RNA, and SMARTer Stranded RNA-Seq kit [Takara, Japan] was used for library construction. Paired-end 75bp sequencing was performed using NextSeq 500/550 v2 kit [Illumina, San Diego, USA].

### 2.3. Differential gene expression analysis

Reads obtained were quality controlled with FastQC and Trimmomatic.^8,9^ Contaminating ribosomal RNA reads were removed using Bowtie2, and reads were then mapped to the human genome sequence database [GRCh38] using STAR and quantified with featureCounts.^10–12^ Genes were filtered, and differential gene expression was analysed based (false discovery rate [FDR] corrected, *p* ≤0.05] using edgeR.^13^ Gene ontology and Kyoto Encyclopaedia of Genes and Genomes [KEGG]/Reactome pathway analysis was conducted using Camera for competitive gene set testing.^14^ ClueGo was used to functionally group gene ontology and pathway annotation networks.^15^

### 2.4. Computational cell deconvolution

xCell, a computational method for cell deconvolution, was used to compare cell types based on genetic signature and to assess for any differences in cell populations derived from mucosal tissue between the three cohorts.^16^

### 2.5. Mucosal immunophenotyping

A collagenase-DNase mix along with gentleMACS Dissociator [Miltenyi Biotec, Germany] was used to digest mucosal samples collected in complete RPMI, followed by gradient centrifugation for lamina propria mononuclear cells [LPMCs] isolation. Panels for CD4 phenotyping and intracellular cytokine staining were analysed. Detailed immunology methods and fluorochrome conjugated antibodies that were used are listed in Supplementary Methods. Stained cells were acquired on BD-Fortessa Flow Cytometer [BD, NJ, USA] and analysed using FlowJo-v10 [Tree Star, OR, USA]. Prism-v8 [Graphpad, California, USA] was used for statistical analysis using Student’s t test.

### 2.6. Mucosally adherent microbial 16S rRNA profiling

Paired DNA extracted as part of the Qiagen AllPrep DNA/RNA Mini Kit was used for 16S rRNA gene amplification and sequencing using the Earth Microbiome Project protocol.^17^ Briefly, 16S rRNA genes were amplified in technical duplicates with primers targeting the 16S rRNA V4 region [515F–806R] using a one-step, single-indexed polymerase chain reaction [PCR] approach. As with DNA/RNA extraction, 16S rRNA gene PCR was done in a batch with appropriate negative controls. Paired-end sequencing [2 × 250bp] was performed on Illumina MiSeq platform [Illumina, San Diego, USA] and processed using Quantitative Insights Into Microbial Ecology 2 [QIIME2] pipeline.^18^ Taxonomy was assigned against the Silva-132–99% OTUs database.^19^ Differences in relative abundance of taxa between cohorts were analysed using linear discriminant analysis [LDA] effect size [LEfSe].^20^ Only taxa with LDA > 2 at a *p*-value <0.05 were considered significant. The functional profiles of microbial communities were inferred using PICRUSt2 derived relative MetaCyc and KEGG pathway analysis and assessed using STAMP.^21,22^

### 2.7. Predictive and network analytics

We used the Random Forest [RF] machine learning ensemble method to obtain predictive performance of features from the transcriptomics, immunophenotype, and 16S rRNA microbial profiling datasets.^23^ Selected genes were mapped to functional information from three databases: IntAct, KEGG, and TRRUST. Network analysis was performed using qgraph package.^24^ A full description and explanation of this methodology are provided in the Supplementary Methods.

## 3. Results

### 3.1. Patient demographics

Thirty patients were recruited into this exploratory study—10 healthy controls, 10 patients with PSC-IBD, and 10 with UC. The patients between the three groups were matched for age and gender ratio. Patients with PSC-IBD and UC were matched for colitis characteristics, with no significant differences in disease extent, Mayo endoscopic sub-score, and medication use. Three patients with PSC-IBD were post-transplant [with recurrence of PSC] and three were on ursodeoxycholic acid [UDCA]. Detailed demographics are shown in Table 1.

**Table 1.** Study cohort demographics.

	HC	PSC-IBD	UC
Age [mean]	47.2 years	42.6 years	39.9 years
Gender M:F	6:4	7:3	7:3
Ulcerative colitis characteristics			
Pan UC		10/10 [100%]	8/10 [80%]
Mayo endoscopic subscore			
0		7/10 [70%]	8/10 [80%]
1		0/10 [0%]	0/10 [0%]
2		1/10 [10%]	2/10 [20%]
3		2/10 [20%]	0/10 [0%]
Drugs			
Proton pump inhibitor	0/10 [0%]	1/10 [10%]	1/10 [10%]
Mesalazine		9/10 [90%]	10/10 [100%]
Azathioprine		1/10 [10%]	2/10[20%]
Vedolizumab		1/10[10%]	0/10[0%]
Other biologics		0/10 [0%]	0/10[0%]
Serum liver tests (median [IQR])			
Albumin [g/L]		48 [5]	46 [2.2]
Bilirubin [µmol/L]		22.5 [24]	13.5 [3.5]
ALT [IU/L]		45.1 [17]	26.1 [5.6]
ALP [IU/L]		181 [178]	74.8 [15.3]
Liver disease characteristics			
Cirrhosis		1/10 [10%]^a^	
On UDCA		3/10 [30%]	
Post-OLT		3/10 [30%]	
Anti-rejection drugs			
Tacrolimus		3/3	
Mycophenolate		1/3	

HC, healthy controls; PSC-IBD, primary sclerosing cholangitis-inflammatory bowel disease ; UC, ulcerative colitis; M, male; F, female; IQR, interquartile range; UDCA, ursodeoxycholic acid; ;ALT, alanine transaminase; ALP, alkaline phosphatase; OLT, orthotopic liver transplant.

^a^Based on ultrasonographic appearances with no complications of portal hypertension [ChildPugh A].

### 3.2. Colonic mucosal transcriptome

#### 3.2.1. Quality control

An average of 38 million PE reads [+/-8.3 million] were generated per sample. Following quality control and in silico decontamination of ribosomal RNA reads, an average of 35 million reads were mapped for subsequent gene expression analysis.

#### 3.2.2. The mucosal transcriptomic landscape is significantly different between PSC-IBD, UC, and HC

**Figure 1.**
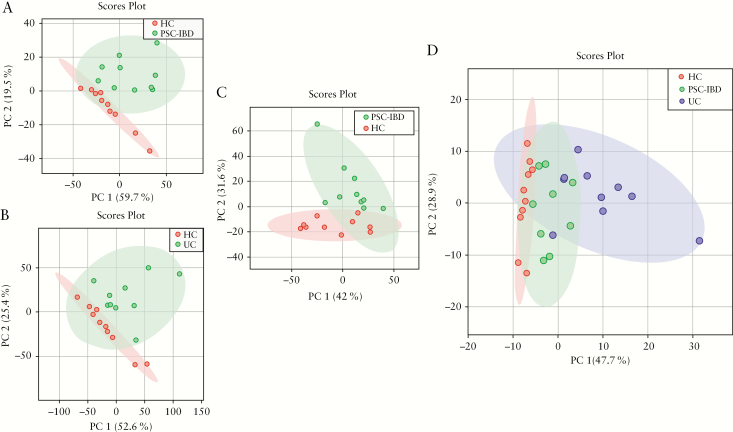
Principal component analysis [PCA] score plot performed on the mucosal transcriptome datasets demonstrating clustering of subjects within, and variation between, cohorts. Dots represents samples and are coloured according to the subject cohort. Ellipse represents 95% confidence. Results are plotted according to the PC1 and PC2 scores, with the percent variation explained by the respective axis. [a] PSC-IBD versus HC; [b] UC versus HC; [c] PSC-UC versus UC; [d] PCA plots demonstrating variation between HC, UC, and PSC-UC samples. The three groups were significantly different from each other [*p* = 0.01]. PSC, primary sclerosing cholangitis; IBD, inflammatory bowel disease; UC, ulcerative colitis; HC, healthy controls.

**Figure 2.**
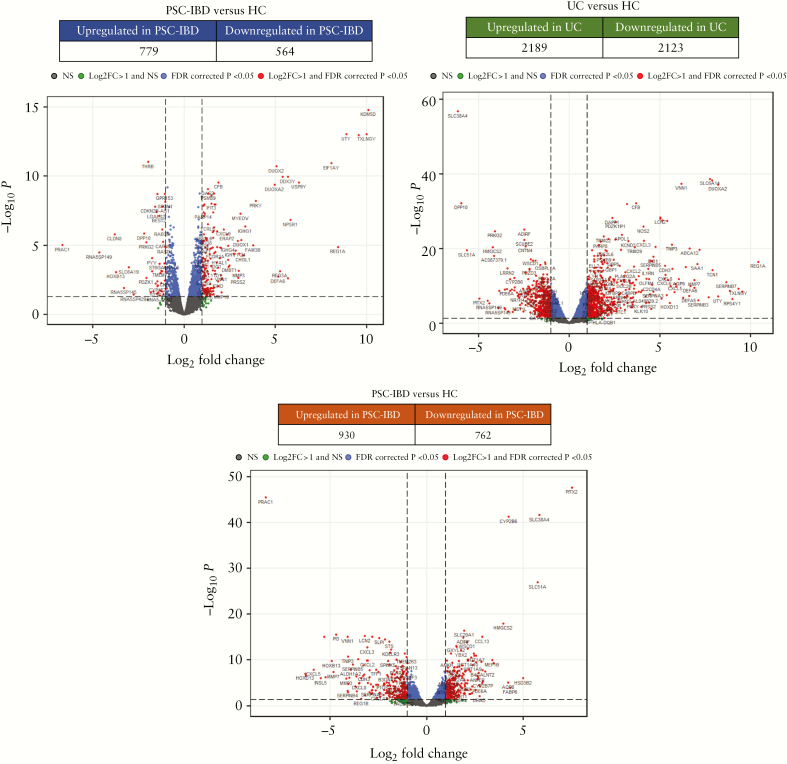
Differential gene expression profiles and volcano plots highlighting differentially expressed genes. [a] PSC-IBD versus HC; [b] UC versus HC; [c] PSC-IBD versus UC. Differentially expressed genes with logFC >1 are shown; 939 genes had shared differential expression in PSC-IBD and UC when compared with healthy controls [588 upregulated; 351 downregulated]. PSC, primary sclerosing cholangitis; IBD, inflammatory bowel disease; UC, ulcerative colitis; HC, healthy controls.

The mucosal transcriptomic profile differed significantly between the three cohorts [*p* = 0.01] as demonstrated by principal component analysis [Figure 1]. In comparison with HC, we found 1343 genes were differentially expressed in PSC-IBD [779 upregulated and 564 downregulated] and 4312 differentially expressed genes in UC [2189 upregulated and 2123 downregulated] [Figure 2a, b]—only 939 genes in PSC-IBD and UC when compared with healthy controls [588 upregulated and 351 downregulated]. On comparing PSC-IBD with UC, 1692 genes were differentially expressed [930 upregulated and 732 downregulated] [Figure 2c]. The full list is provided as Supplementary File 1, available as Supplementary data at *ECCO-JCC* online.

### 3.3. Pathway analysis

We performed pathway analysis to assess for enrichment of particular gene annotation categories amongst the differential gene expression datasets [full list provided as Supplementary File 2, available as Supplementary data at *ECCO-JCC* online].

#### 3.3.1. PSC-IBD compared with HC

Gene ontology analysis revealed enrichment of 948 biological processes in PSC-IBD compared with HC. Of these, 824 were upregulated and were primarily associated with innate, adaptive, and humoral immune response [Supplementary Figure 2a, available as Supplementary data at *ECCO-JCC* online]. Furthermore, there was upregulation of anti-microbial defense and extracellular matrix remodelling processes. Similarly, analysis using KEGG/Reactome revealed that 186 pathways that were differentially regulated [130 upregulated] in PSC-IBD compared with HC and associated with various immunological mechanisms [Supplementary Figure 3, available as Supplementary data at *ECCO-JCC* online].

#### 3.3.2. UC compared with HC

Pathway analysis of UC compared with HC demonstrated that 982 gene ontology biological processes were significantly enriched [837 upregulated]. KEGG/Reactome analysis showed that 253 pathways were significantly enriched [186 upregulated]. Similar to the pathway comparison between PSC-IBD and HC, there was upregulation in pathways associated with a multitude of facets of immunological response, antimicrobial defense, and extracellular matrix remodelling [Supplementary Figure 2b]. Downregulated pathways were associated with metabolic processes, cellular respiration, and butyrate metabolism [Supplementary Figure 4, available as Supplementary data at *ECCO-JCC* online].

#### 3.3.3. PSC-IBD compared with UC

**Figure 3.**
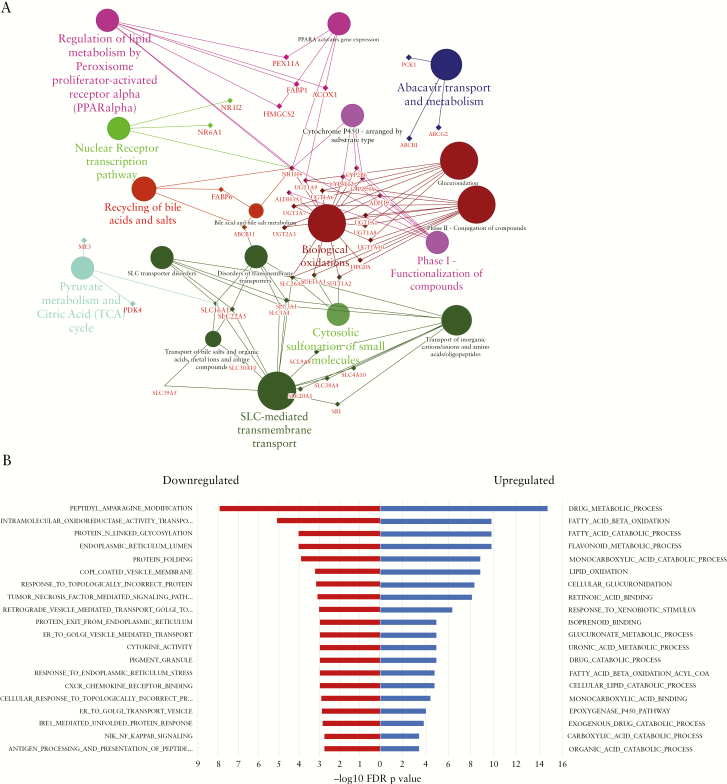
Bioinformatic analysis identifies pathways including bile acid signalling as relevant to disease distinctions. [a] Detailed enrichment analysis of gene clusters in functionally grouped network. Gene networks and pathways involving key nuclear receptors involved in bile acid homeostasis such as FXR, PPAR, conjugation, binding, and transport are significantly upregulated in PSC-IBD compared with controls; [b] top 20 gene ontology biological processes PSC-IBD vs UC demonstrate metabolic pathways, many of which are involved in bile acid homeostasis are upregulated in PSC-IBD compared with UC, whereas immune activation and defense pathways are upregulated in UC compared with PSC-IBD. PSC, primary sclerosing cholangitis; IBD, inflammatory bowel disease; UC, ulcerative colitis; HC.

Gene ontology analysis comparing PSC-IBD with UC demonstrated significant enrichment of 563 biological processes. Of these, 104 were upregulated in PSC-IBD and were associated with fatty acid metabolic processes, glucuronidation, bile acid and bile salt metabolism processes, and transport. Processes such as those associated with immunological response were downregulated compared with UC. KEGG/Reactome analysis revealed differential regulation of 238 pathways in PSC-IBD compared with UC [62 upregulated] with findings similar to gene ontology analysis. Additionally, pathways associated with bile acid homeostasis including PPAR signalling, nuclear receptor transcription, and bile acid recycling, were upregulated in PSC-IBD compared with UC [Figure 3]. Pathways associated with DNA damage response, telomere maintenance, transition of cell cycle phases, and cell replication were downregulated in PSC-IBD.

We explored the effects of specific confounders on DEGs in PSC-IBD. In patients with a liver transplant [*n* = 3] who were also on tacrolimus, only REG3A, DEFA5, DEFA6, SNORA66, and PRSS2 were upregulated and VSIG4 downregulated. In patients with UDCA [*n* = 3], only CYP3A4 was differentially expressed [upregulated]. There were no significant DEGs associated with use of mycophenolote [*n* = 1], biologics [*n* = 1], or cirrhosis [*n* = 1]. Furthermore, to explore confounding effects of liver transplantation, we conducted a subgroup analysis of pre-liver transplant PSC-IBD patients [*n* = 7]. This revealed that the changes in bile acid homeostatic and immunological pathways were similar in comparison with the PSC-IBD cohort that included three post-liver transplant patients with recurrence of PSC [Supplementary Results, available as Supplementary data at *ECCO-JCC* online].

### 3.4. Cell deconvolution

Computational cell deconvolution showed that only the dendritic cell subset was increased in PSC-IBD compared with HC [*p* = 0.03, mean delta = 0.004; Supplementary Figure 5, available as Supplementary data at *ECCO-JCC* online]. There were no other differences in proportions of epithelial cells or immune subsets between the cohorts.

### 3.5. Mucosal immunophenotyping

Representative gating strategy is shown in Supplementary Figure 6, available as Supplementary data at *ECCO-JCC* online. PSC-IBD and UC were both characterised by a significantly higher frequency of colonic mucosal CCR6 + CD161 + Th17 cells compared with HC [18.62% vs 8.41%, *p* <0.001; and 15.61% vs 8.41%, *p* = 0.04, respectively]. CCR6-CXCR3 + CCR5 + Th1 cells were significantly lower in PSC-IBD compared with HC [15.38% vs 23.62% respectively, *p* <0.01] [Supplementary Figure 7, available as Supplementary data at *ECCO-JCC* online]. No differences in Th1 populations were seen between UC and HC. Significantly increased frequencies of IL17-producing CD4 T cells were observed in both PSC-IBD and UC compared with HC [8.48% vs 5.67%, *p* <0.01; and 8.8% vs 5.67%, *p* = 0.03, respectively]. There were no differences identified in Th2, Tregs, TNF-alpha, and IFN-gamma producing CD4 cells between the disease cohorts and healthy controls. There were no significant differences in any of the immunological subsets between PSC-IBD and UC.

### 3.6. 16S rRNA sequencing

#### 3.6.1. Gut microbial profiles differ significantly between the three groups

**Figure 4.**
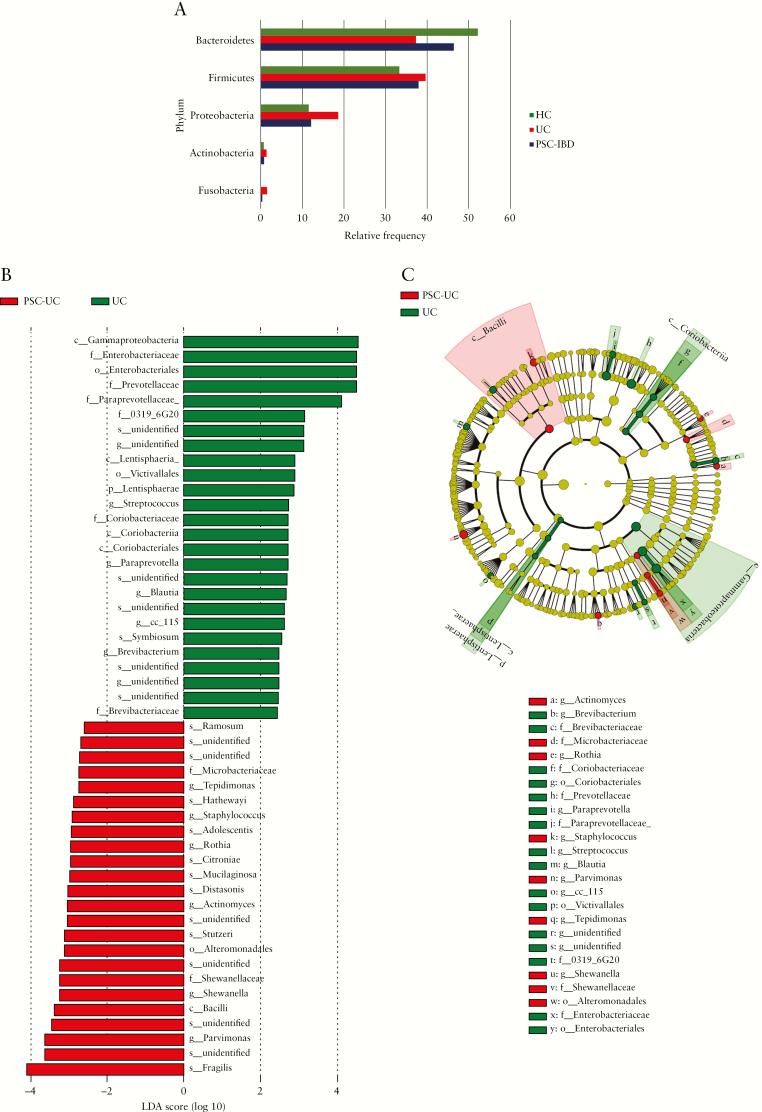
Distinct microbiota taxa in patients with PSC-IBD. [a] Phylum level differences in the three cohorts—UC and PSC-IBD are characterised by a relative expansion of Proteobacteria and Fusobacteria and reduction of Bacteroidetes compared with HC; [b] microbial taxa comparing PSC-IBD and UC. Association of specific microbiota taxa with PSC-UC and UC by linear discriminant analysis [LDA] effect size [LEfSe]. Red indicates taxa enriched in PSC-IBD and green indicates taxa enriched in UC; [c] Cladogram representation of the gut microbial taxa associated with PSC-IBD and UC. PSC-IBD is associated with an increased abundance of bacteria expressing bile salt hydrolase and hydroxysteroid dehydrogenases [such as *Bacteroides fragilis*, *Roseburia spp*, *Shewanella spp*.,and *Clostridium ramosum*] in comparison with UC. PSC, primary sclerosing cholangitis; IBD, inflammatory bowel disease; UC, ulcerative colitis.

Totals of 6.6 million reads [110 035 reads/sample] and 3396 features were obtained after quality control. No significant differences in alpha diversity were observed [Supplementary Figure 8, available as Supplementary data at *ECCO-JCC* online]. The microbial profiles of the three groups were significantly different based on their beta-diversity [*p* = 0.01], as shown in Supplementary Figure 9, available as Supplementary data at *ECCO-JCC* online. Additionally, the colitis phenotype [PSC-IBD and UC together] was significantly different to HC [*p* = 0.007]. UC was characterised by a relative expansion of the phyla Proteobacteria and Fusobacteria along with reduction of phyla Bacteroidetes, compared with HC [Figure 4a]. PSC-IBD in comparison with HC was associated with significant shifts in taxa which included a reduction in family Lachnospiraceae and increase in class Bacilli, genus *Pseudomonas* and *Streptoccocus*, and species *Haemophilus parainfluenzae* [Supplementary Figure 10b, available as Supplementary data at *ECCO-JCC* online]. UC was associated with shifts that included significant reductions in *Ruminoccocus* species and increases in genus *Anaerococcus* compared with HC [Supplementary Figure 10a, available as Supplementary data at *ECCO-JCC* online]. In comparison with UC, PSC-IBD was characterised by a significant difference in 50 taxa, of which 24 were enriched in PSC-IBD. In PSC-IBD there were reductions in taxa that included phylum Lentisphaerae, class Gammaproteobacteria, families Enterobacteriacea, Prevotellacae, Paraprevotellacae, and Myxococcales, and genus *Streptococcus*. PSC-IBD was associated with a significant increase in taxa that included the class Bacilli, genus *Staphylococcus*, and species *Parvimonas* sp. and *Bacteroides fragilis*. [Figure 4b, c].

#### 3.6.2. Predicted metagenomic pathways in PSC-IBD compared with UC

**Figure 5.**
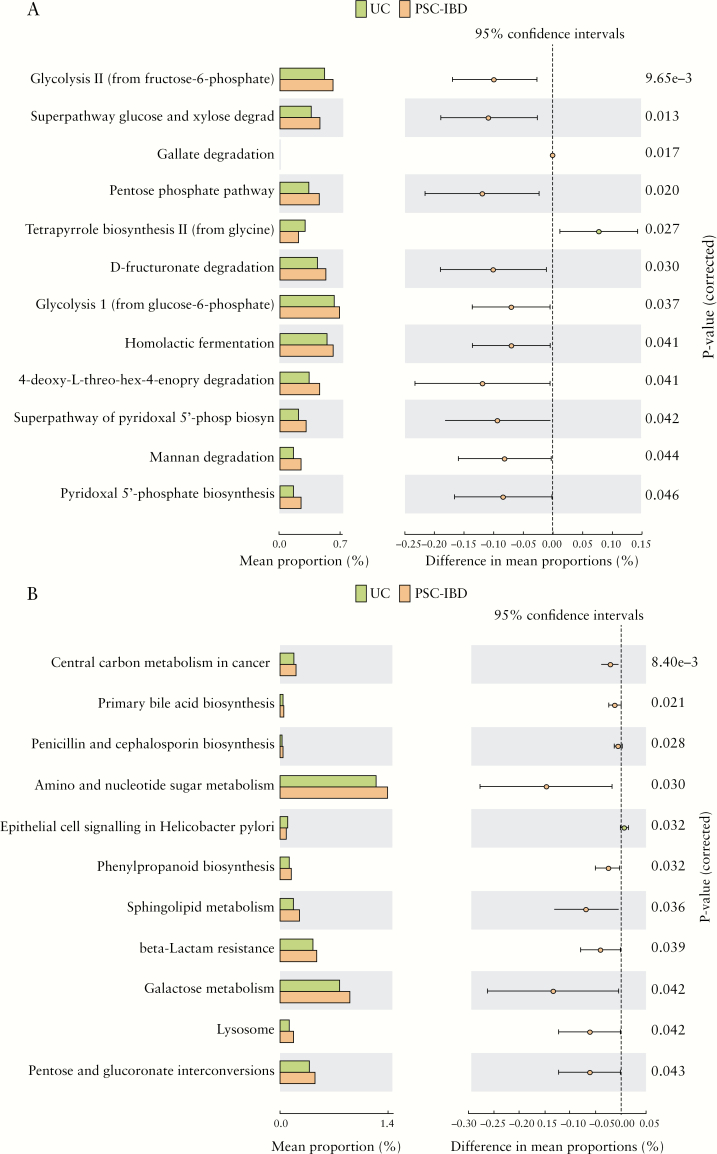
Functional classification of the predicted metagenome content of the microbiota of PSC-IBD compared with UC. [a] MetaCyc pathways; and [b] KEGG pathways. There is significant enrichment of primary bile acid biosynthesis [associated with significantly higher expression of bile salt hydrolase and hydroxysteroid dehydrogenases], pentose and glucoronate interconversions, and galactose metabolism in PSC-IBD compared with UC. PSC, primary sclerosing cholangitis; IBD, inflammatory bowel disease; UC, ulcerative colitis.

Metacyc pathway analysis of the microbiome functions inferred from 16S rRNA gene sequence profiles revealed an increase in pathways such as glycolysis and mannan degradation. The predicted KEGG pathways significantly enriched in PSC-IBD compared with UC included primary bile acid biosynthesis [associated with significantly higher expression of bile salt hydrolase and hydroxysteroid dehydrogenases] and pentose and glucoronate interconversions. [Figure 5]’

### 3.7. Predictive analytics for discrimination of disease states and data integration for mapping biological interactions

#### 3.7.1. Predicting disease modelling and regulatory network analysis of gene expression

In PSC-IBD vs HC samples, based on the area under the curve [AUC], the ‘top 25’ genes from RNA seq data with AUC value 0.97 and confidence interval [CI] of 0.80–1 were identified. Similarly, for UC vs HC samples, the AUC value was 0.97 with 15 genes and the CI was 0.77–1. In the case of PSC-IBD vs UC, the top 50 genes resulted in an AUC value of 0.96 [CI 0.66–1]. We identified the *STOM* gene to have maximum connections in PSC-IBD vs HC; *LYN* gene for UC vs HC, and *TUBB2A* gene for PSC-IBD vs UC. The full list of genes can be found in Supplementary Table 3, available as Supplementary data at *ECCO-JCC* online.

#### 3.7.2. Integrating transcriptomics, immunophenotype, and 16S rRNA microbial profile

**Figure 6.**
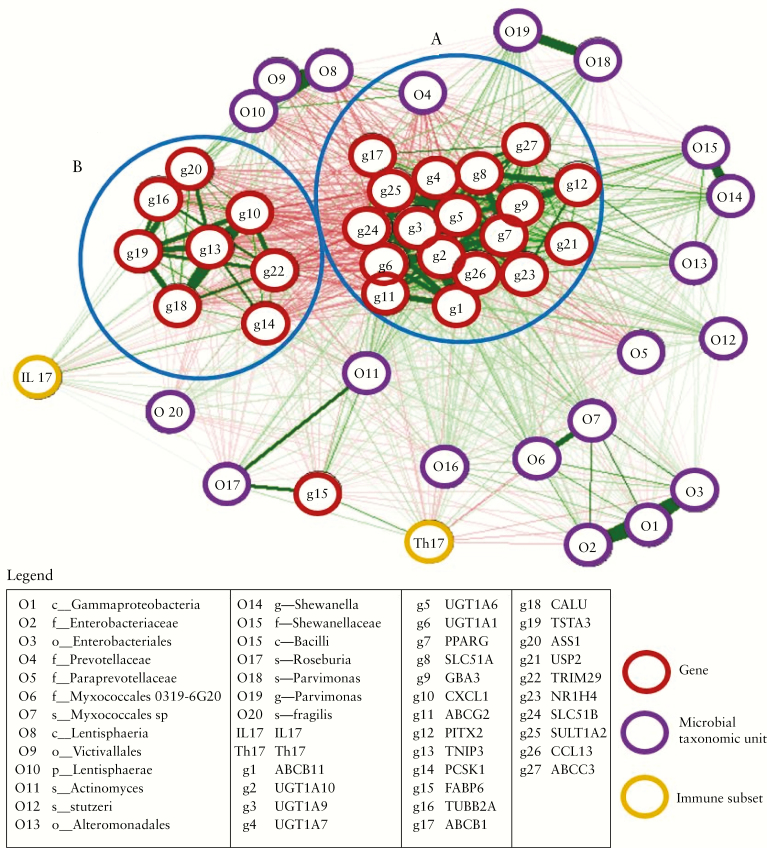
Correlation networks between the mucosal transcriptome, 16S microbial profiles, and immunophenotype in patients with PSC-IBD. The mucosal transcriptome is in red, 16S microbial profiles purple. Genes were selected based on supervised and unsupervised dimension reduction as described in Methods. Microbial taxa were selected based on the loading values more than log2[3] values. Th17- and IL17-expressing cells were selected as they were significantly upregulated in PSC-IBD and UC compared with HC. Red line indicates negative correlation and green positive correlation. Thickness of the line shows strength of the correlation between each features. Clusters are defined based on a high inter-feature correlation [r = 0.8]. Two main clusters were identified—cluster A consists of genes involved in bile acid homeostasis; cluster B demonstrates genes associated with cancer regulatory pathways. PSC, primary sclerosing cholangitis; IBD, inflammatory bowel disease; UC, ulcerative colitis; HC, healthy controls.

Genes differentially expressed between PSC-IBD and UC were selected based on AUC values, maximum connectivity, and biological relevance. 16S rRNA microbial profiling was selected based on linear discriminant analysis [LDA] effect size [LEfSe] more than log[10] value of greater than or less than 3, between PSC-IBD and UC. Th17 and IL17 were selected as they were significantly different in PSC-IBD and UC compared with HC. As shown in Figure 6, through multi-omics integration analysis we observed two network clusters, involving genes associated with bile acid homeostasis and genes associated with cancer regulatory pathways, which were significant between PSC-IBD and UC.

## 4. Discussion

It is well recognised by clinicians that the clinical phenotype of PSC-IBD is different from UC. This, along with differences in genetic risk, collectively support the prediction that biological pathways related to disease behaviour should differ. Our work previously demonstrated that the mucosally adherent gut microbial profiles were different between patients with PSC-IBD, those with UC, and healthy controls. Our current study presents data from an independent cohort of patients to explore the gene expression, immunophenotype, and microbial profiles in the colonic mucosa. Using an integrative systems biology approach in this pilot study, we demonstrate significant differences between the colonic mucosal landscape in patients with PSC-IBD and UC, thereby highlighting opportunities to harness distinctions in biology to inform future therapy.

Our comparative analysis of mucosal transcriptome in colitic patients and healthy controls confirms that the colonic phenotype in both PSC-IBD and UC is primarily immune-mediated, with upregulation of pathways driving multiple facets of the innate, adaptive, and humoral immune response. Furthermore, genes and pathways associated with recognised biological mechanisms, including anti-microbial defense response and extracellular matrix remodelling, are also differentially upregulated in comparison with healthy controls. In our transcriptomic analysis, PSC-IBD had very distinct mucosal transcriptomic profile compared with UC. Only 939 genes had shared differential expression in PSC-IBD and UC compared with healthy controls. These genes amount to 70% of DEGs between PSC-IBD and HC and 22% of DEGs between UC and HC. Importantly, 1692 genes were differentially expressed between PSC-IBD and UC. Analysis of the genes and pathways associated with this differential expression highlights key differences in physiological processes, with enrichment of biological processes involved in bile acid homeostasis, glucuronidation, and specific metabolic functions in PSC-IBD compared with UC.^25^ Bile acid regulation is mediated through a negative feedback mechanism as part of its enterohepatic recirculation, by hormones such as fibroblast growth factor [FGF] 15 [murine] and 19 [human]. Bile acids are reabsorbed primarily by terminal ileal enterocytes and partly by colonocytes. This activates FXR [farnesoid X receptor] by upregulation of the FGF15/19 pathway, which consequently leads to inhibition of hepatic bile acids synthesis through suppression of CYP7A1 enzyme. We have shown that multiple nuclear receptors, specifically FXR [*NR1H4*], PXR [pregnane X receptor], and PPAR-γ [peroxisome proliferator-activated receptor gamma], are upregulated in PSC-IBD compared with UC. As bile acids are inherently cytotoxic, these nuclear receptors—in particular FXR, a bile acid-activated transcriptional factor—may be activated in order to induce gene expression circuitry to protect against bile acid toxicity, by shutting down expression of genes that increase influx and synthesis of bile. The intracellular concentration of bile acids is an important determinant of FXR transcriptional activity. Multiple isoforms of UDP-glucuronosyltransferases [UGT] and sulphonyltransferases [SULTS] and *GBA3* [glucosylceramidase beta 3] were equally found to be significantly upregulated in PSC-IBD compared with UC. Unconjugated hydrophobic dihydroxy bile acids that are passively absorbed in the distal ileum and colon are converted to more hydrophilic and less toxic conjugated derivatives in response to FXR and PPAR by UGT [glucuronidation], SULT [sulphation], and GBA [hydrolysis] and exported across the basolateral membrane.^26–28^ We have shown that gene expression of FXR and PPAR dependent ileal bile acid binding protein [IBAP] and multiple basolateral bile acid efflux transporters, including OSTα/OSTβ [organic solute transporter alpha-beta], *BSEP* [ABCB11, bile salt export pump], *BCRP* [ABCG2, breast cancer resistance protein], and *MRP3* [ABCC3, Multidrug Resistance-Associated Protein 3], are significantly upregulated in PSC-IBD compared with UC. The position of BSEP is unclear, with low levels of hepatic expression being described.^29^ Although the expression of FGF19, a key hormone produced in response of FXR activation, was unchanged, the MEP1β gene [meprin A subunit-β] produced in response to FGF19 was highly upregulated in PSC-IBD.^30^ This FXR/PPAR-mediated protective positive feedback effect, possibly as a consequence of increased intracellular bile acid concentrations, functions to accelerate neutralisation, binding, and its elimination in order to reduce intracellular colonic bile acid toxicity.^27,31,32^ It has been shown that the faecal bile acid pool is significantly reduced in PSC-IBD, and it is unclear whether this is due to reduced delivery into the colon related either to cholestasis or increased bile acid clearance.^2^

In this study, we have confirmed from mucosal analysis previous findings by our group and others,^2–4^ that the gut microbiota profile is different between PSC-IBD, UC, and HC. The dysbiosis seen in PSC-IBD appears to be associated with changes in bile acid metabolic pathways, which is congruent with our transcriptomic and pathway analysis. Patients with PSC-IBD had significantly higher abundances of the species *Bacteroides fragilis*, *Roseburia spp.*, *Shewanella sp.p* and *Clostridium ramosum* compared with UC patients. These bacteria express bile salt hydrolase [BSH], an enzyme that catalyses the deconjugation of bile acids in the gut.^33^ These bacteria have also been shown to express hydroxysteroid dehydrogenases that are involved in biotransformation of primary to secondary bile acids.^34^ Inferred metagenomics corroborated enrichment of BSH and hydroxysteroid dehydrogenases in PSC-IBD gut microbiota compared with UC [and HC] in our dataset. Vancomycin, an antibiotic that specifically targets Gram-positive bacteria [many of which are involved in dehydoxylation of primary bile acids into secondary bile acids] has been shown to induce remission of colitis in patients with PSC-IBD.^35^ Furthermore, amine oxidase-expressing bacteria, *Sphingomonas* sp., were found to be upregulated in PSC-IBD compared with UC. This enzyme is associated with aberrant homing of gut lymphocytes to the liver, a proposed mechanism underlying the PSC-IBD gut-liver inflammatory axis.^36^

Our study demonstrates for the first time that the colonic mucosal immune response in PSC-IBD is characterised by a significantly higher Th17 cell and IL-17-producing CD4 T cell population compared with HC. These findings were similar to those seen in the UC cohort; however, patients with PSC-IBD also have a lower Th1 and higher IL17/IFNγ-producing CD4 cell population compared with controls. IL17-producing Th17 cells are often present at sites of chronic tissue inflammation in multiple autoimmune diseases.^37^ Th17 cells are critical drivers of inflammation in autoimmune diseases, and are likely to be induced by specific components of gut microbiota.^38^ The role of bile acids in directly mediating host immunity by controlling Th17 response has been shown to be associated with Th17 expansion and IL17 production.^39^ We found significant upregulation of CYP27A1 expression in PSC-IBD compared with UC. This enzyme generates oxysterols, which are key intermediates for bile acid synthesis and function as RORγt ligands to drive Th17 differentiation.^40^

Genes and pathways associated with cancer regulation, including DNA damage repair and checkpoints, p53 signalling, mitosis transition, and APC/CCdc20 mediated Cyclin A degradation, were significantly downregulated in PSC-IBD compared with UC. Network analysis revealed the gene *TUBB2A* to be a potential key mediator in PSC-IBD compared with UC. We have previously shown methylation of a component of this gene [*TUBB6*] significantly positively correlated colonic dysplasia.^41^ Interestingly, p-ANCAs in autoimmune liver diseases are reported to be directed against a further component of the beta-tubulin family, *TUBB5*, which cross-reacts with the bacterial protein FtsZ, reflecting an abnormal immune response to gut bacteria.^42^ PRAC1 gene was found to be 8 log-fold downregulated in PSC-IBD, and expression of this gene is associated with reduced susceptibility for right-sided cancers.^43^

One of the major strengths of our study is that this is the first multi-omics to date which has attempted to unravel disease mechanisms by integrating mucosal transcriptomics, immunophenotyping, and mucosal microbial profiling. Through this approach, we have demonstrated that consistent with clinical phenotype and GWAS datasets, the biological mechanisms underlying colonic pathology appear to be distinct in PSC-IBD compared with UC. We have made novel observations and proposed disease processes that need validation through e -vivo experiments. Through cell deconvolution, we were able to ascertain that the transcriptomic findings were not a result of significant differences in cell subset populations apart from a slight increase in dendritic cells in PSC-IBD compared with HC.

This technique does have limitations, and future studies should strongly consider using single cell RNA-sequencing. Moreover, a significant number of genes that we have explored are likely to have multiple unknown or less established functions, thereby limiting the pathway analysis approach. Power calculations for RNA-sequencing experiments are not fully established; however, between six and 12 biological replicates have been recommended.^44^ Nevertheless, we accept that a lack of validation cohort as part of this analysis is a limitation of this study. We adopted strict exclusion criteria to address potential confounders and, despite our relatively small sample size, we were able to infer significant biological differences with high confidence between the three cohorts, including through predictive modelling.^45^ As shown in Table 1 patients with PSC-IBD and UC were matched for IBD characteristics. All patients with PSC-IBD and eight patients with UC had pancolitis [and the remaining two had left-sided colitis]. Therefore, by analysing biopsies taken from the sigmoid colon, we attempted to control for biological changes associated with anatomical location and inflammation. Furthermore, in our previous study we found that the microbial profiles did not differ between different segments of the colon within patients with PSC-IBD.^4^

Our data are pilot in nature and we must recognise the challenge of confounding from clinical heterogeneity. We did not find specific confounders, which included liver transplantation and immunosuppression including biologics, to alter differential gene expression results, in particular bile acid signalling genes. Through a subgroup analysis of pre-transplant PSC-IBD patients, as outlined in Supplementary Results, we were able to demonstrate that the key findings of changes in bile acid homeostatic and immunological pathways were not different in comparison with the PSC-IBD cohort that included three post-liver transplant patients with recurrence of PSC. However, we appreciate that these subgroup analyses are limited by the sample size. We recognise that UCDA use in three patients with PSC-IBD can modulate intestinal bile acid homeostatic pathways; however, only the CYP3A4 gene was differentially regulated in PSC-IBD. Furthermore, UDCA has not been shown to activate FXR expression, but rather may antagonise it as demonstrated by a pharmacodynamic study and FXR binding experiments.^46^ It is therefore seems unlikely for UDCA to have had an impact on the gene expression profiles seen in PSC-IBD. Although the median bilirubin in PSC-IBD was 24.5μmmol/L, only two patients were jaundiced [bilirubin <80μmmol/L] one of whom had Child_Pugh A cirrhosis with no differential gene expression as a consequence of this. We also recognise that cause or effect of cholestasis would be difficult to establish from our data, as the very nature of PSC means cholestasis is its primary manifestation. However, identifying a control arm that consisted of UDCA-naïve patients with cholestatic liver disease and without colonic inflammation, undergoing colonoscopy, was in essence not feasible for this pilot dataset. Furthermore, it would be important to validate these findings through an independent cohort along with targeted gene expression analysis using a qPCR-based or 3’-seq-based approach. Finally, it remains to be established how the colon compares with ileal bile acid homeostatic mechanisms in health and disease, and the role of diet as a modifier of the PSC-IBD transcriptome, epigenome, and microbiome. Future studies should strongly consider dietary evaluation alongside investigating tissue, faecal, and serum bile acid profiles.

In conclusion, by using a systems biology approach, we demonstrate that the colonic inflammation in PSC-IBD, like UC, is immune-mediated when compared with healthy controls, and presents as a predominant Th17- and IL17-producing CD4 cell response. PSC-IBD, however, is transcriptomically distinct from UC, with dysregulation of genes associated with multiple bile acid homeostatic pathways, potentially mediated by the corresponding differences in gut dysbiosis demonstrated between the IBD cohorts studied. These findings support the hypothesis that colonic mucosal immune-mediated inflammation in PSC-IBD is contributed to by colonic mucosal bile acid toxicity. Further work is required to understand whether the dysregulated bile acid metabolism in PSC-IBD is driven by the gut microbiota or vice versa, and how host genetics plays into this interaction. This preliminary work thus informs potential future therapeutic approaches for PSC-IBD that include novel bile acid/microbial manipulation.

## Supplementary Material

jjaa021_suppl_Supplementary_Figure_1

jjaa021_suppl_Supplementary_Figure_2

jjaa021_suppl_Supplementary_Figure_3

jjaa021_suppl_Supplementary_Figure_4

jjaa021_suppl_Supplementary_Figure_5

jjaa021_suppl_Supplementary_Figure_6

jjaa021_suppl_Supplementary_Figure_7

jjaa021_suppl_Supplementary_Figure_8

jjaa021_suppl_Supplementary_Figure_9

jjaa021_suppl_Supplementary_Figure_10

jjaa021_suppl_Supplementary_Table_1

jjaa021_suppl_Supplementary_Figure_Legends

jjaa021_suppl_Supplementary_Table_2

jjaa021_suppl_Supplementary_Methods

jjaa021_suppl_Supplementary_Results

jjaa021_suppl_Supplementary_Table_3

jjaa021_suppl_Supplementary_Table_4

## Data Availability

RNA-Seq data for this study have been deposited in ArrayExpress database with the accession code E-MTAB-7915. 16S rRNA data have been submitted to the Sequence Read Archive [SRA] under accession reference PRJNA533720.

## References

[1] Hirschfield GM, Karlsen TH, Lindor KD, Adams DH Primary sclerosing cholangitis. Lancet 2013;382:1587–99. 23810223 10.1016/S0140-6736(13)60096-3

[2] Torres J, Palmela C, Brito H, et al. The gut microbiota, bile acids and their correlation in primary sclerosing cholangitis associated with inflammatory bowel disease. United European Gastroenterol J 2018;6:112–22. 10.1177/2050640617708953 PMC5802676 29435321

[3] Kummen M, Holm K, Anmarkrud JA, et al. The gut microbial profile in patients with primary sclerosing cholangitis is distinct from patients with ulcerative colitis without biliary disease and healthy controls. Gut 2017;66:611–9. 26887816 10.1136/gutjnl-2015-310500

[4] Quraishi MN, Sergeant M, Kay G, et al. The gut-adherent microbiota of PSC-IBD is distinct to that of IBD. Gut 2017;66:386–8. 10.1136/gutjnl-2016-311915 27196590

[5] Ji SG, Juran BD, Mucha S, et al.; UK-PSC Consortium; International IBD Genetics Consortium; International PSC Study Group Genome-wide association study of primary sclerosing cholangitis identifies new risk loci and quantifies the genetic relationship with inflammatory bowel disease. Nat Genet 2017;49:269–73. 27992413 10.1038/ng.3745 PMC5540332

[6] Ellinghaus D, Folseraas T, Holm K, et al. Genome-wide association analysis in primary sclerosing cholangitis and ulcerative colitis identifies risk loci at GPR35 and TCF4. Hepatology 2013;58:1074–83. 22821403 10.1002/hep.25977

[7] Liu JZ, Hov JR, Folseraas T, et al.; UK-PSCSC Consortium; International PSC Study Group; International IBD Genetics Consortium Dense genotyping of immune-related disease regions identifies nine new risk loci for primary sclerosing cholangitis. Nat Genet 2013;45:670–5. 23603763 10.1038/ng.2616 PMC3667736

[8] Andrews S FastQC: a quality control tool for high throughput sequence data 2010.http://www.bioinformatics.babraham.ac.uk/projects/fastqc. Accessed December 12, 2018.

[9] Bolger AM, Lohse M, Usadel B Trimmomatic: a flexible trimmer for Illumina sequence data. Bioinformatics 2014;30:2114–20. 24695404 10.1093/bioinformatics/btu170 PMC4103590

[10] Langmead B, Salzberg SL Fast gapped-read alignment with Bowtie 2. Nat Methods 2012;9:357–9. 22388286 10.1038/nmeth.1923 PMC3322381

[11] Dobin A, Davis CA, Schlesinger F, et al. STAR: ultrafast universal RNA-seq aligner. Bioinformatics 2013;29:15–21. 23104886 10.1093/bioinformatics/bts635 PMC3530905

[12] Liao Y, Smyth GK, Shi W featureCounts: an efficient general purpose program for assigning sequence reads to genomic features. Bioinformatics 2014;30:923–30. 24227677 10.1093/bioinformatics/btt656

[13] Robinson MD, McCarthy DJ, Smyth GK edgeR: a Bioconductor package for differential expression analysis of digital gene expression data. Bioinformatics 2010;26:139–40. 19910308 10.1093/bioinformatics/btp616 PMC2796818

[14] Wu D, Smyth GK Camera: a competitive gene set test accounting for inter-gene correlation. Nucleic Acids Res 2012;40:e133. 22638577 10.1093/nar/gks461 PMC3458527

[15] Bindea G, Mlecnik B, Hackl H, et al. ClueGO: a Cytoscape plug-in to decipher functionally grouped gene ontology and pathway annotation networks. Bioinformatics 2009;25:1091–3. 19237447 10.1093/bioinformatics/btp101 PMC2666812

[16] Aran D, Hu Z, Butte AJ xCell: digitally portraying the tissue cellular heterogeneity landscape. Genome Biol 2017;18:220. 29141660 10.1186/s13059-017-1349-1 PMC5688663

[17] Earth Microbiome Project. Illumina 16s PCR protocols http://www.earthmicrobiome.org/protocols-and-standards/. Accessed January 18, 2019.

[18] Bolyen E, Rideout JR, Dillon MR, et al. Reproducible, interactive, scalable and extensible microbiome data science using QIIME 2. Nat Biotechnol 2019;37:852–857. 10.1038/s41587-019-0209-9 PMC7015180 31341288

[19] Quast C, Pruesse E, Yilmaz P, et al. The SILVA ribosomal RNA gene database project: improved data processing and web-based tools. Nucleic Acids Res 2013;41:D590–6. 23193283 10.1093/nar/gks1219 PMC3531112

[20] Segata N, Izard J, Waldron L, et al. Metagenomic biomarker discovery and explanation. Genome Biol 2011;12:R60. 21702898 10.1186/gb-2011-12-6-r60 PMC3218848

[21] Langille MGI Exploring linkages between taxonomic and functional profiles of the human microbiome. mSystems 2018;3. 10.1128/mSystems.00163-17 PMC5881027 29629420

[22] Parks DH, Tyson GW, Hugenholtz P, Beiko RG STAMP: statistical analysis of taxonomic and functional profiles. Bioinformatics 2014;30:3123–4. 25061070 10.1093/bioinformatics/btu494 PMC4609014

[23] Breiman L Random forests. Machine Learning 2001;45:5–32.

[24] Epskamp S, Cramer AOJ, Waldorp LJ, Schmittmann VD, Borsboom D qgraph: Network visualizations of relationships in psychometric data. J Stat Softw 2012;48:1–18.

[25] Chen ML, Takeda K, Sundrud MS Emerging roles of bile acids in mucosal immunity and inflammation. Mucosal Immunol 2019;1 10.1038/s41385-019-0162-4. 30952999

[26] Zelcer N, Saeki T, Bot I, Kuil A, Borst P Transport of bile acids in multidrug-resistance-protein 3-overexpressing cells co-transfected with the ileal Na+-dependent bile-acid transporter. Biochem J 2003;369:23–30. 12220224 10.1042/BJ20021081 PMC1223054

[27] Zhou X, Cao L, Jiang C, et al. PPARα-UGT axis activation represses intestinal FXR-FGF15 feedback signalling and exacerbates experimental colitis. Nat Commun 2014;5:4573. 10.1038/ncomms5573 PMC4164778 25183423

[28] Strassburg CP, Nguyen N, Manns MP, Tukey RH UDP-glucuronosyltransferase activity in human liver and colon. Gastroenterology 1999;116:149–60. 9869613 10.1016/s0016-5085(99)70239-8

[29] Hilgendorf C, Ahlin G, Seithel A, Artursson P, Ungell AL, Karlsson J Expression of thirty-six drug transporter genes in human intestine, liver, kidney, and organotypic cell lines. Drug Metab Dispos 2007;35:1333–40. 17496207 10.1124/dmd.107.014902

[30] Becker-Pauly C, Barré O, Schilling O, et al. Proteomic analyses reveal an acidic prime side specificity for the astacin metalloprotease family reflected by physiological substrates. Mol Cell Proteomics 2011;10:M111.009233. 10.1074/mcp.M111.009233 PMC3186203 21693781

[31] Pineda Torra I, Claudel T, Duval C, Kosykh V, Fruchart JC, Staels B Bile acids induce the expression of the human peroxisome proliferator-activated receptor alpha gene via activation of the farnesoid X receptor. Mol Endocrinol 2003;17:259–72. 12554753 10.1210/me.2002-0120

[32] Dawson PA Roles of Ileal ASBT and OSTα-OSTβ in Regulating Bile Acid Signaling. Dig Dis 2017;35:261–6. 28249269 10.1159/000450987 PMC5432121

[33] Song Z, Cai Y, Lao X, et al. Taxonomic profiling and populational patterns of bacterial bile salt hydrolase [BSH] genes based on worldwide human gut microbiome. Microbiome 2019;7:9. 30674356 10.1186/s40168-019-0628-3 PMC6345003

[34] Fukiya S, Arata M, Kawashima H, et al. Conversion of cholic acid and chenodeoxycholic acid into their 7-oxo derivatives by Bacteroides intestinalis AM-1 isolated from human feces. FEMS Microbiol Lett 2009;293:263–70. 19243441 10.1111/j.1574-6968.2009.01531.x

[35] Dao A, Abidian M, Lestrange A, Mattar M, Rangnekar A, Charabaty A. Oral Vancomycin Induces and Maintains Remission of Ulcerative Colitis in the Subset of Patients With Associated Primary Sclerosing Cholangitis *|* Inflammatory Bowel Diseases. Oxford, UK: Oxford Academic. 2019;25:90–e91. 10.1093/ibd/izz027 30838381

[36] Trivedi PJ, Tickle J, Vesterhus MN, et al. Vascular adhesion protein-1 is elevated in primary sclerosing cholangitis, is predictive of clinical outcome and facilitates recruitment of gut-tropic lymphocytes to liver in a substrate-dependent manner. Gut 2018;67:1135–45. 28428344 10.1136/gutjnl-2016-312354 PMC5969351

[37] Burkett PR, Meyer zu Horste G, Kuchroo VK Pouring fuel on the fire: Th17 cells, the environment, and autoimmunity. J Clin Invest 2015;125:2211–9. 25961452 10.1172/JCI78085 PMC4497747

[38] Britton GJ, Contijoch EJ, Mogno I, et al. Microbiotas from humans with inflammatory bowel disease alter the balance of gut Th17 and RORγt+ regulatory T Cells and exacerbate colitis in mice. Immunity 2019;50:212–24.e4. 30650377 10.1016/j.immuni.2018.12.015 PMC6512335

[39] Hang S, Paik D, Yao L, et al. Bile acid metabolites control TH17 and Treg cell differentiation. Nature 2019;576:143–8. 31776512 10.1038/s41586-019-1785-z PMC6949019

[40] Soroosh P, Wu J, Xue X, et al. Oxysterols are agonist ligands of RORγt and drive Th17 cell differentiation. Proc Natl Acad Sci U S A 2014;111:12163–8. 25092323 10.1073/pnas.1322807111 PMC4143045

[41] Beggs AD, James J, Caldwell G, et al. Discovery and validation of methylation biomarkers for ulcerative colitis associated neoplasia. Inflamm Bowel Dis 2018;24:1503–9. 29762666 10.1093/ibd/izy119 PMC6176894

[42] Terjung B, Söhne J, Lechtenberg B, et al. p-ANCAs in autoimmune liver disorders recognise human beta-tubulin isotype 5 and cross-react with microbial protein FtsZ. Gut 2010;59:808–16. 19951907 10.1136/gut.2008.157818

[43] Hu W, Yang Y, Li X, et al. Multi-omics approach reveals distinct differences in left- and right-sided colon cancer. Mol Cancer Res 2018;16:476–85. 29187560 10.1158/1541-7786.MCR-17-0483

[44] Poplawski A, Binder H Feasibility of sample size calculation for RNA-seq studies. Brief Bioinform 2018;19:713–20. 28100468 10.1093/bib/bbw144

[45] Acharjee A, Ament Z, West JA, Stanley E, Griffin JL Integration of metabolomics, lipidomics and clinical data using a machine learning method. BMC Bioinformatics 2016;17:440. 28185575 10.1186/s12859-016-1292-2 PMC5133491

[46] Mueller M, Thorell A, Claudel T, et al. Ursodeoxycholic acid exerts farnesoid X receptor-antagonistic effects on bile acid and lipid metabolism in morbid obesity. J Hepatol 2015;62:1398–404. 25617503 10.1016/j.jhep.2014.12.034 PMC4451470

