# Supplementary figures and images for: A Pilot Integrative Analysis of Colonic Gene Expression, Gut Microbiota, and Immune Infiltration in Primary Sclerosing Cholangitis-Inflammatory Bowel Disease: Association of Disease With Bile Acid Pathways
Source: J Crohns Colitis. 2020 Feb 4;14(7):935–47. doi: 10.1093/ecco-jcc/jjaa021 (PMC7392170; doi:10.1093/ecco-jcc/jjaa021)

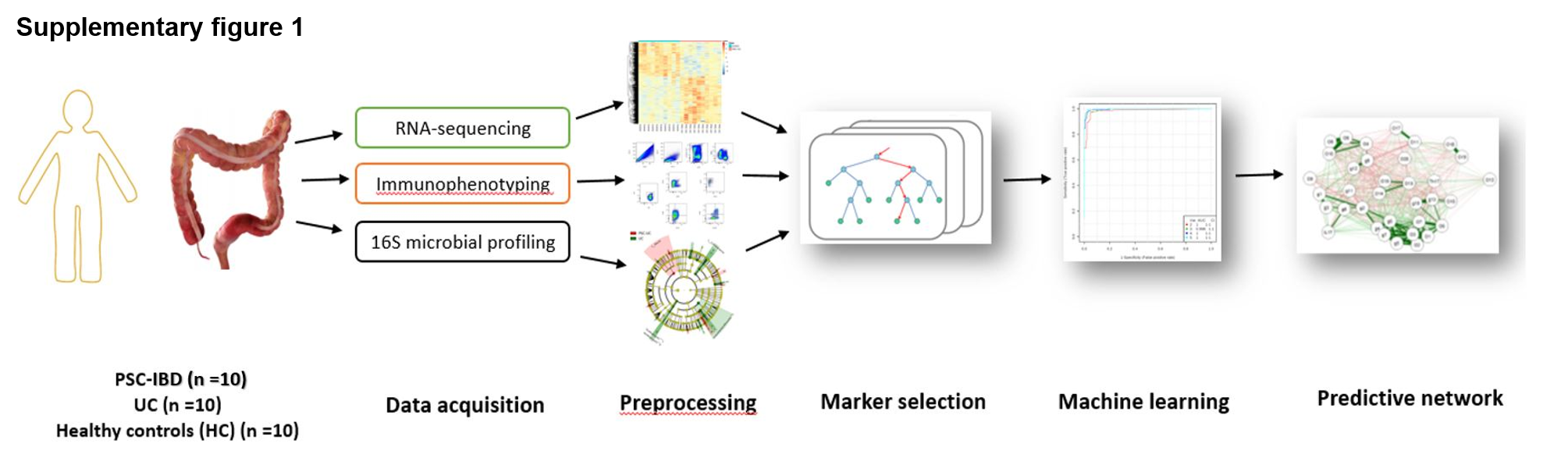

Supplement: jjaa021_suppl_Supplementary_Figure_1 [file jjaa021_suppl_supplementary_figure_1.png]

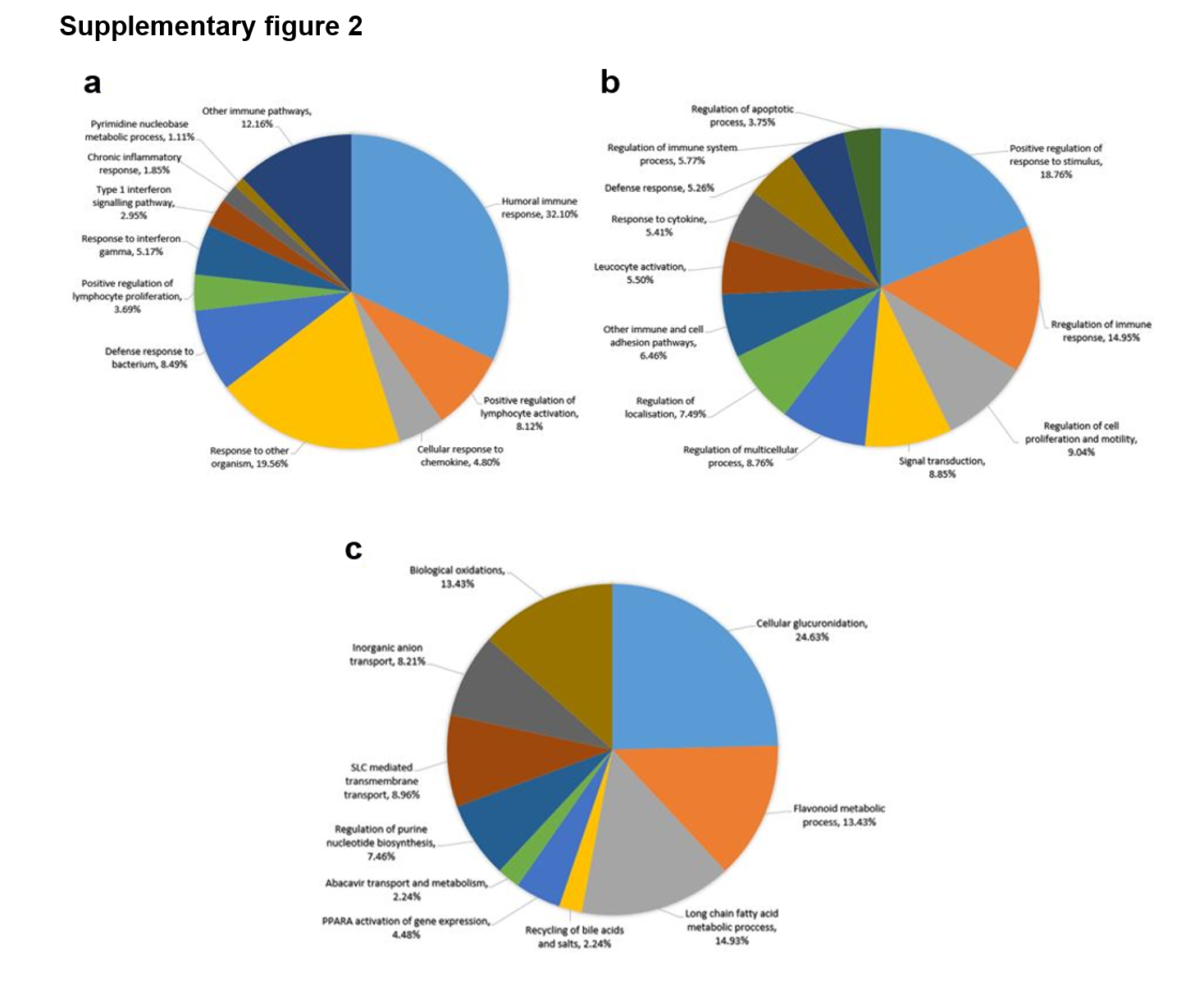

Supplement: jjaa021_suppl_Supplementary_Figure_2 [file jjaa021_suppl_supplementary_figure_2.png]

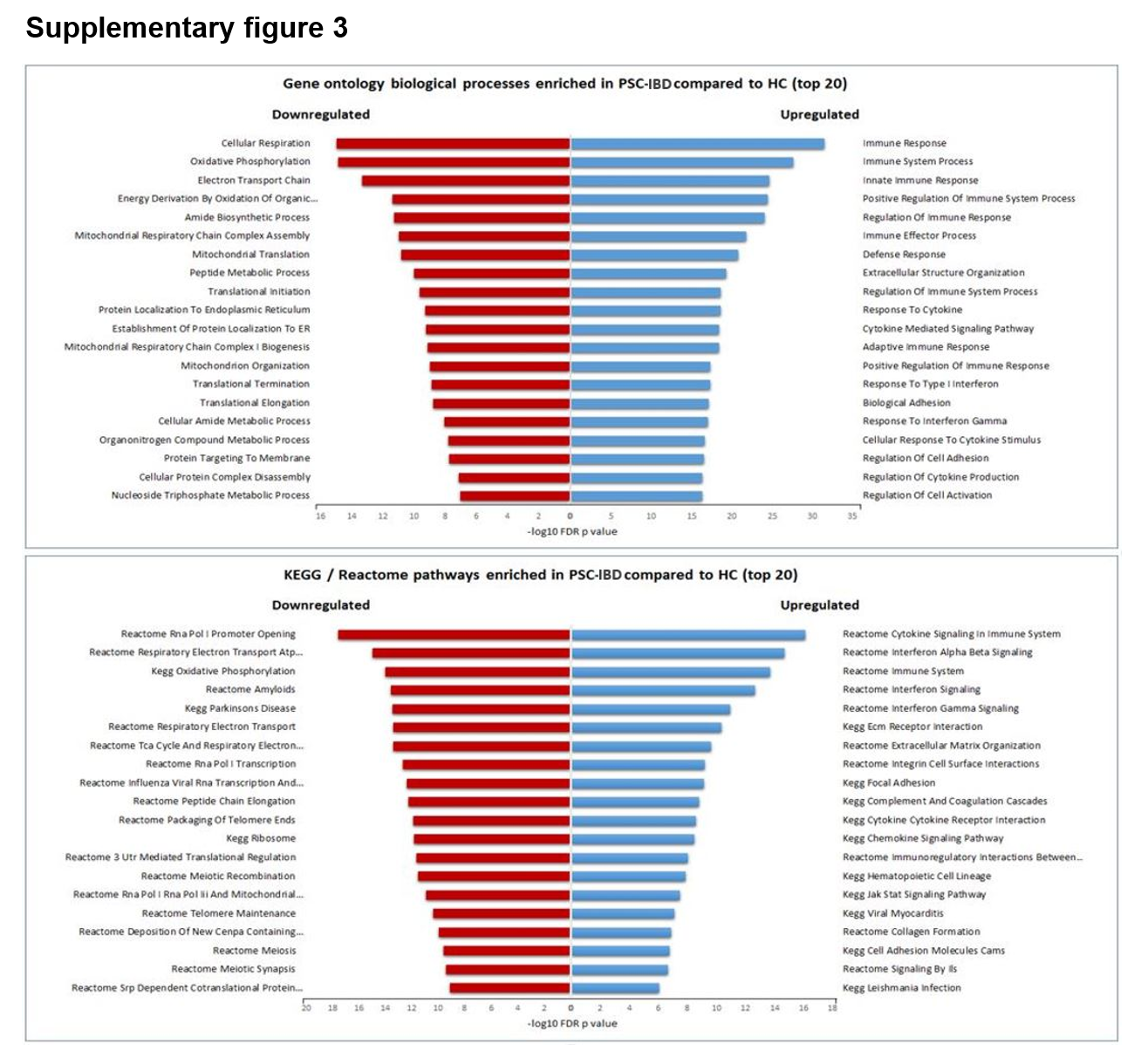

Supplement: jjaa021_suppl_Supplementary_Figure_3 [file jjaa021_suppl_supplementary_figure_3.png]

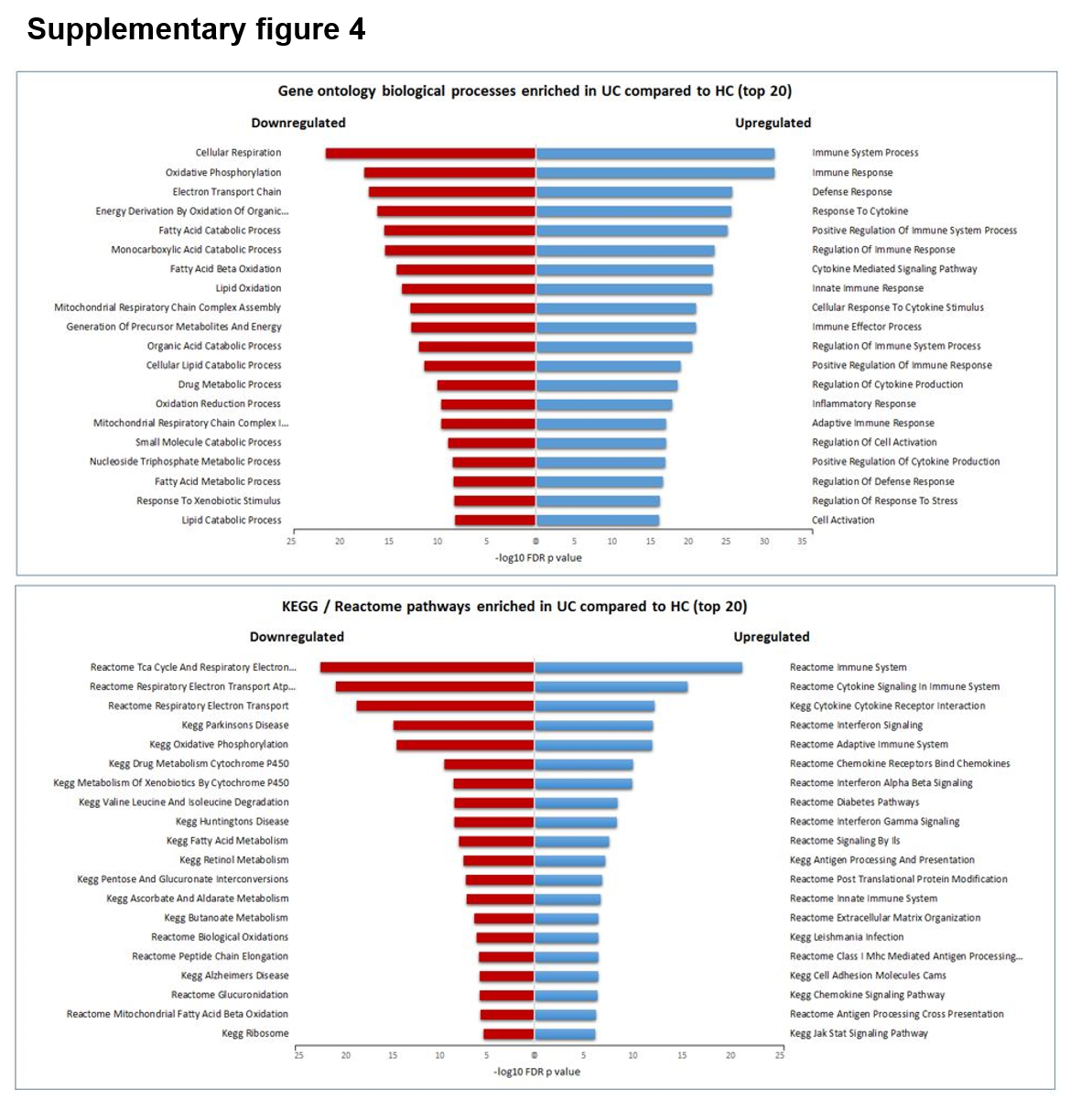

Supplement: jjaa021_suppl_Supplementary_Figure_4 [file jjaa021_suppl_supplementary_figure_4.png]

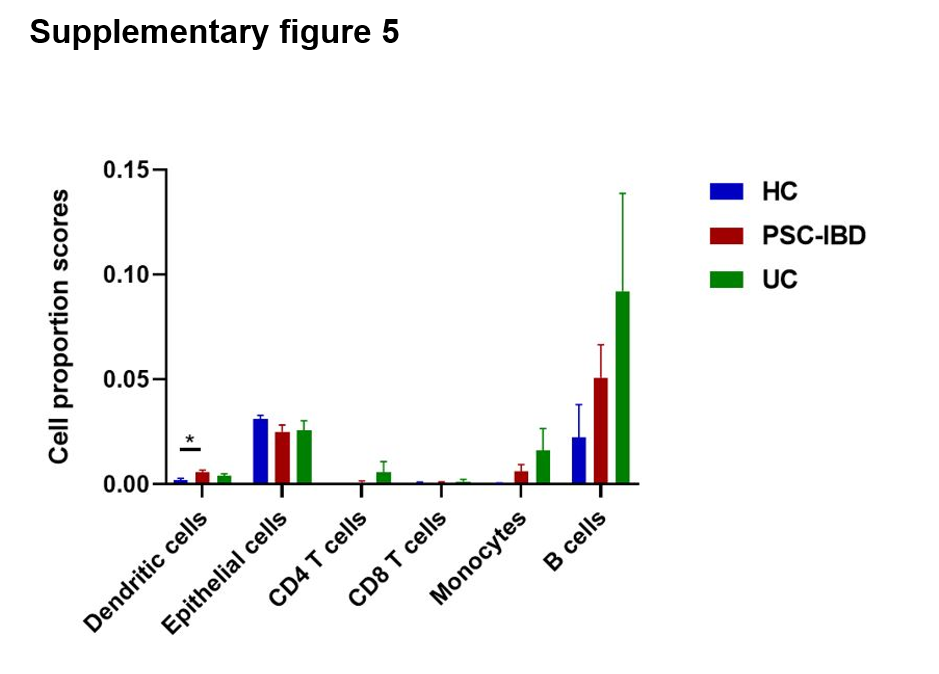

Supplement: jjaa021_suppl_Supplementary_Figure_5 [file jjaa021_suppl_supplementary_figure_5.png]

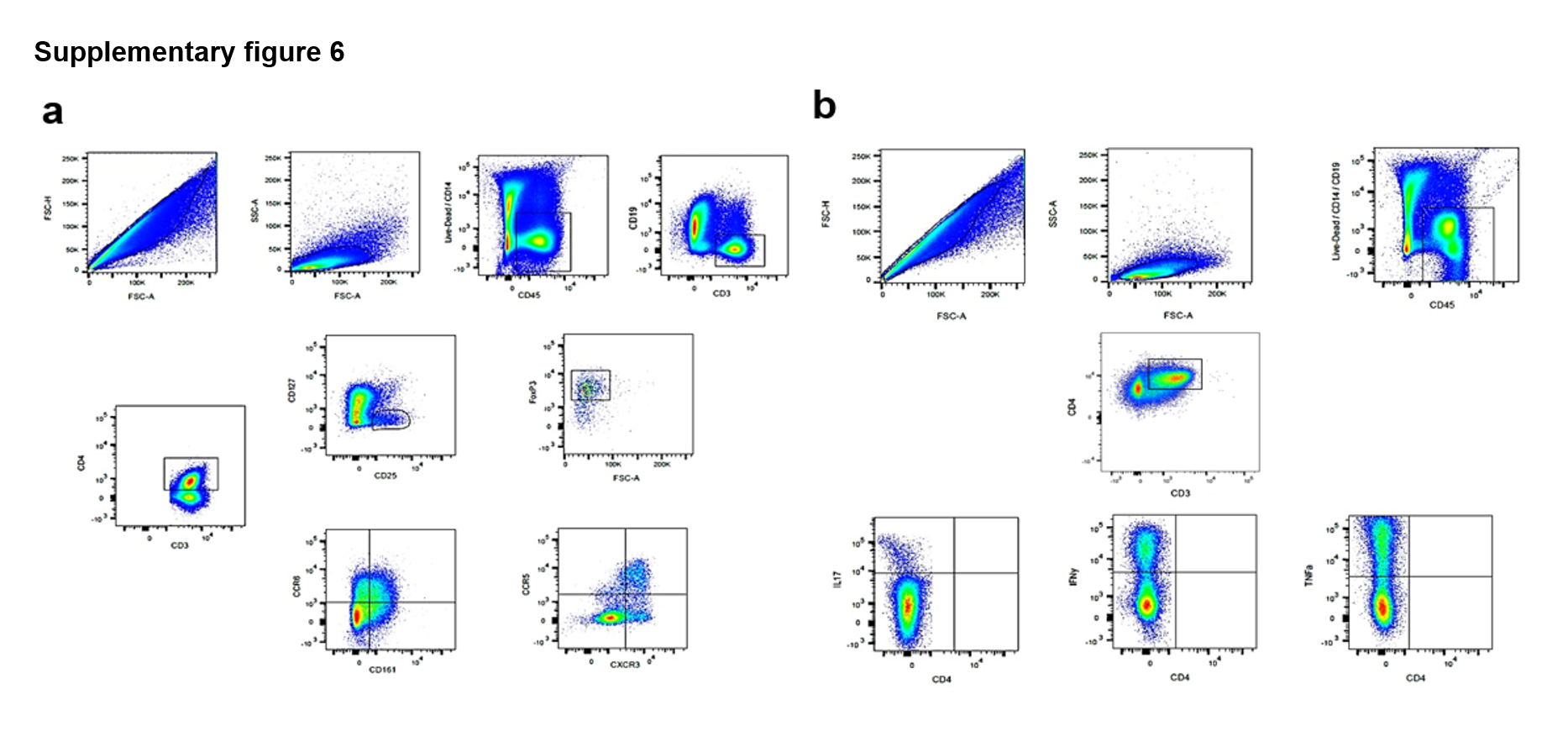

Supplement: jjaa021_suppl_Supplementary_Figure_6 [file jjaa021_suppl_supplementary_figure_6.png]

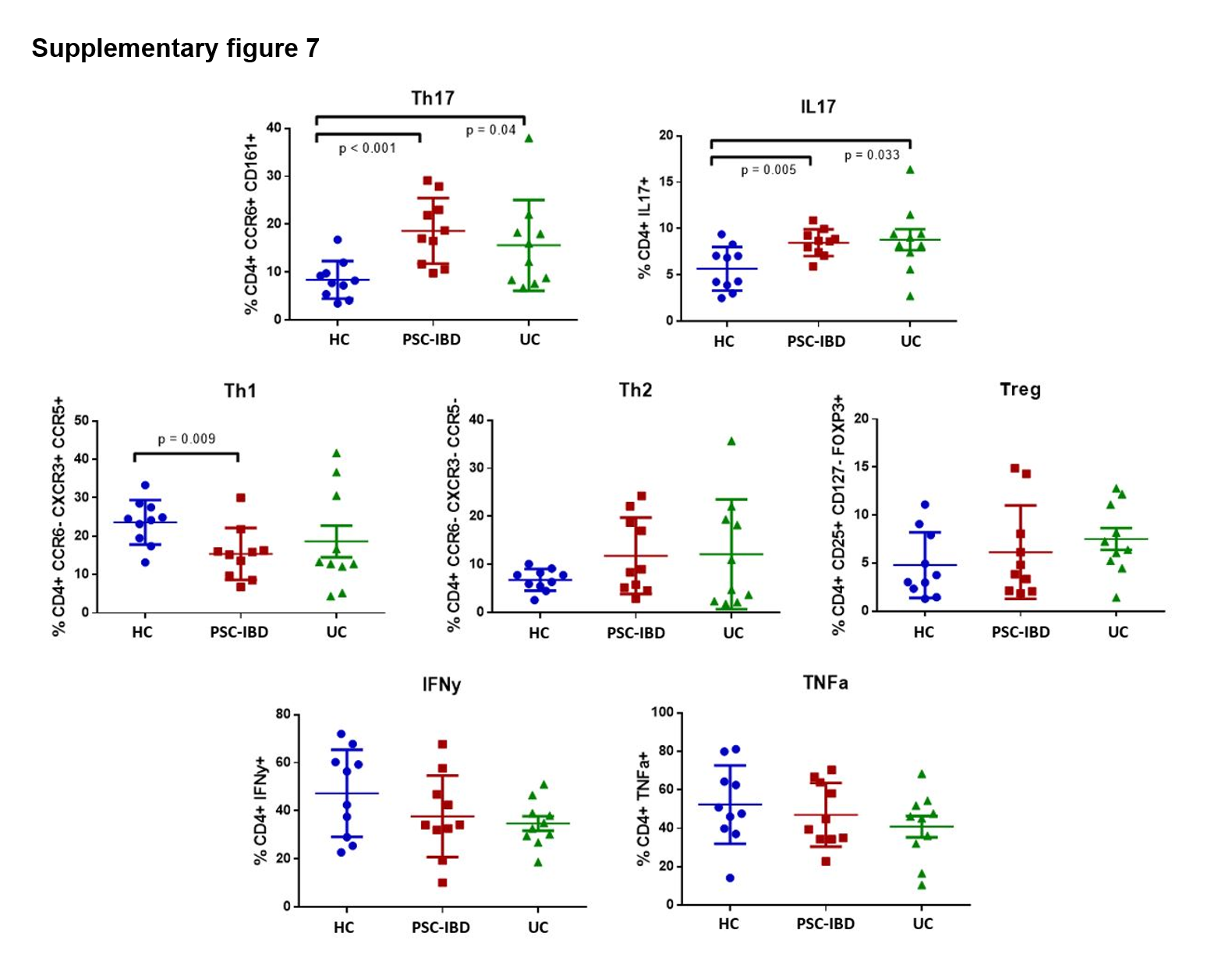

Supplement: jjaa021_suppl_Supplementary_Figure_7 [file jjaa021_suppl_supplementary_figure_7.png]

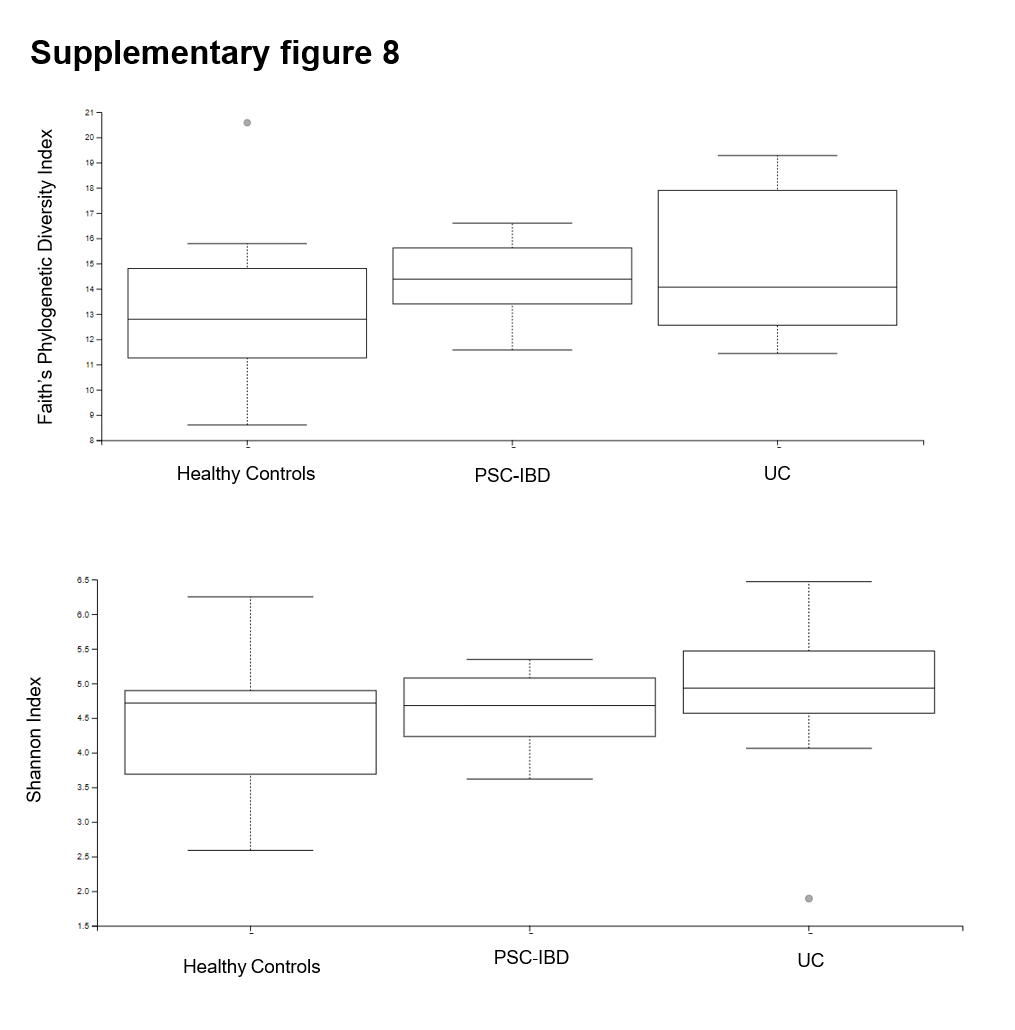

Supplement: jjaa021_suppl_Supplementary_Figure_8 [file jjaa021_suppl_supplementary_figure_8.png]

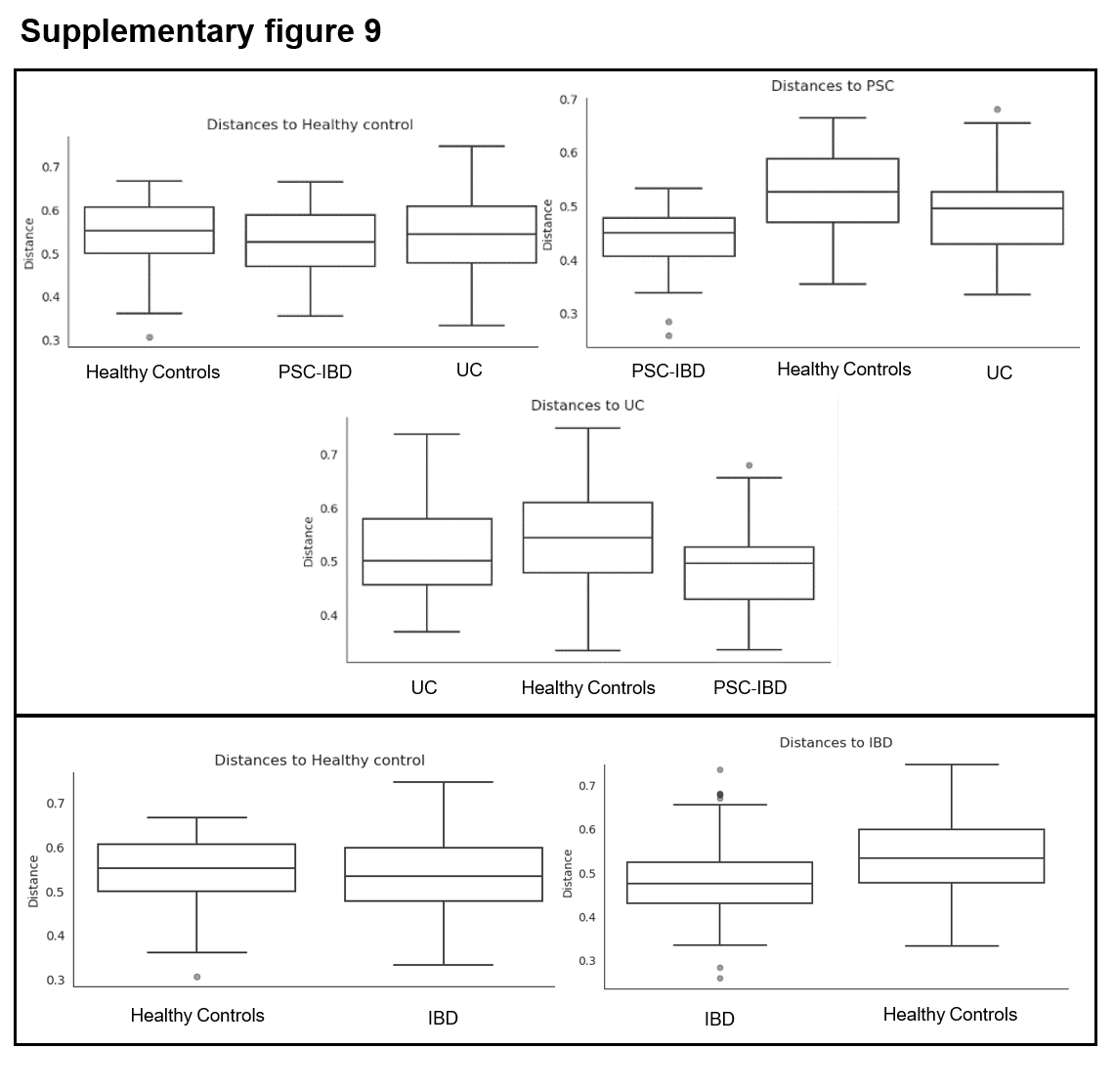

Supplement: jjaa021_suppl_Supplementary_Figure_9 [file jjaa021_suppl_supplementary_figure_9.png]

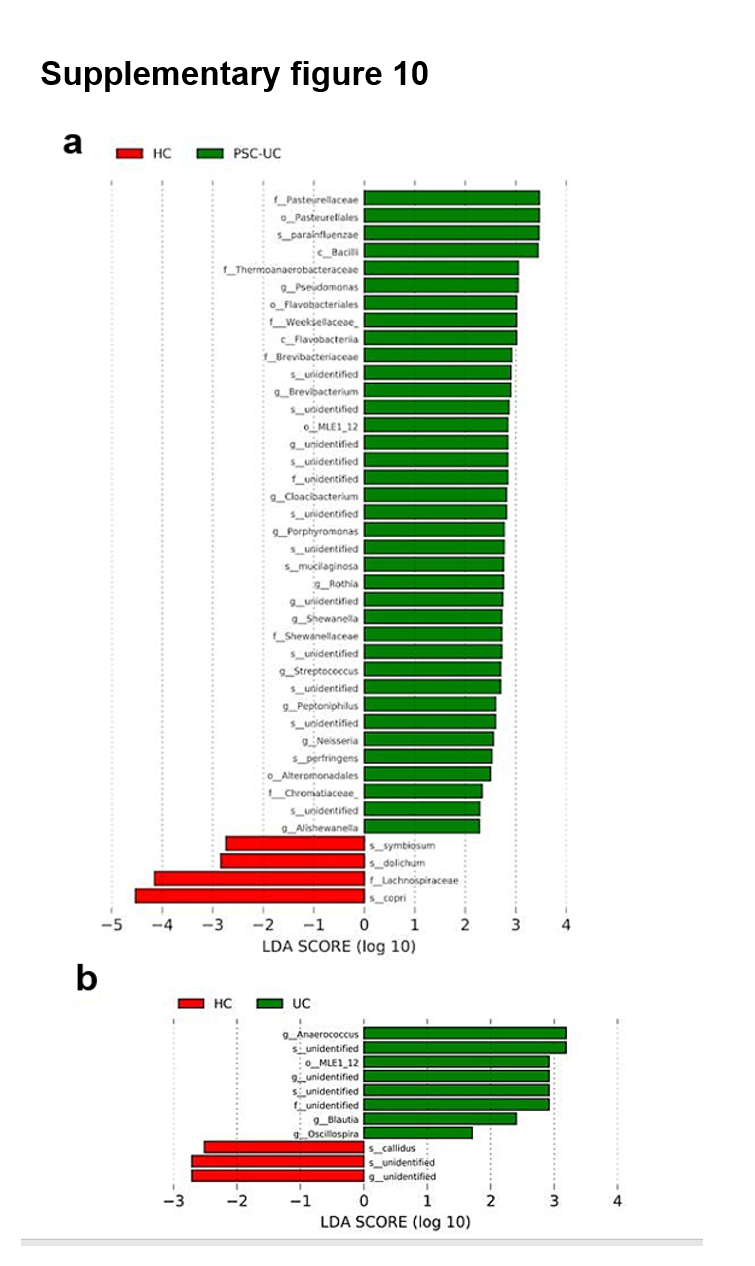

Supplement: jjaa021_suppl_Supplementary_Figure_10 [file jjaa021_suppl_supplementary_figure_10.png]
