# Supplementary material for: A Pilot Integrative Analysis of Colonic Gene Expression, Gut Microbiota, and Immune Infiltration in Primary Sclerosing Cholangitis-Inflammatory Bowel Disease: Association of Disease With Bile Acid Pathways
Source: J Crohns Colitis. 2020 Feb 4;14(7):935–47. doi: 10.1093/ecco-jcc/jjaa021 (PMC7392170; doi:10.1093/ecco-jcc/jjaa021)
Supplement: jjaa021_suppl_Supplementary_Figure_Legends [file jjaa021_suppl_supplementary_figure_legends.docx]

**Supplementary figure legends**

**Supplementary figure 1** Overview of data analysis. Colonic biopsies taken from ten patients with PSC-IBD, UC and healthy controls (HC) were analysed for gene expression profiles by RNA-sequencing, immunophenotype and 16S microbial profile. Features / markers were selected based on supervised and unsupervised machine learning methods for multi-omics integration.

**Supplementary figure 2** Functional annotation enrichment analyses using ClueGO charts of upregulated genes using predefined gene ontology biological processes (log2FC >1, FDR P value < 0.05) in **(a)** in PSC-UC compared to HC and **(b)** in UC compared to HC and **(c)** in PSC-IBD compared to UC. Pathways involved in host immune response and microbial defence are upregulated in both PSC-IBD and UC when compared to healthy controls. In contrast, multiple pathways involved in bile acid homeostasis and transport are upregulated in PSC-IBD when compared to UC.

**Supplementary figure 3** Top 20 gene ontology biological processes and KEGG/Reactome pathways by competitive gene set testing using Camera demonstrate upregulation of immune mediated pathways in PSC-IBD compared to HC.

**Supplementary figure 4** Top 20 gene ontology biological processes and KEGG/Reactome pathways by competitive gene set testing using Camera demonstrate upregulation of immune mediated pathways in UC compared to HC.

**Supplementary figure 5** Computational cell deconvolution was performed to estimate the relative composition of immune cell subsets and epithelial cells in each sample. Cell subset proportion scores are generated based on the xCell pipeline’s gene signature based method. This demonstrated only an increase in dendritic cell population in PSC-IBD compared to controls (P=0.03).

**Supplementary figure 6** Representative gating strategy for mucosal immunophenotyping analysis. A) CD4 subsets. FMO for CD4 and CCR6 used to define gates. B) Intracellular cytokine producing CD4 cells. FMO used to define gates on cytokine expression**.**

**Supplementary figure 7** Scatterplot of paired mucosal immune subsets in PSC-IBD, UC and HC. Bars denote standard error of mean. Probability values (P values) provided for significant subset comparisons. Both Th17 cells (CD4+CCR6+CD161+) and IL17 producing CD4 cells (CD4+IL17+) were significantly increased in PSC-UC and UC compared to healthy controlled. Additionally Th1 cells (CD4+CCR6-CD161-CCR5+CXCR3+) were decreased in PSC-IBD only compared to healthy controls. No changes in were identified in regulatory T cell population (CD4+CD127-CD25+FoxP3+) or in Th2 like CD4 subsets (CD4+CCR6-CD161-CCR5-CXCR3-) in either PSC-IBD or UC compared to controls. There were no differences identified between PSC-IBD and UC in any of the subsets.

**Supplementary figure 8** Alpha diversity analysis comparing cohorts. As estimated by Shannon and Faith’s diversity index analysis demonstrated no significant difference in richness and diversity between the three cohorts.

**Supplementary figure 9** Beta diversity analysis comparing cohorts. Unweighted Unifrac diversity boxplots comparing beta diversity of gut microbiota between (a) three cohorts (b) Healthy controls and IBD (PSC-IBD and UC). The microbial profiles of the three groups were significantly different to each other based on their beta-diversity (p=0.01). Additionally, the colitis phenotype (PSC-IBD and UC) was significantly different to HC (p=0.007).

**Supplementary figure 10** Association of specific microbiota taxa as determined by by linear discriminant analysis (LDA) effect size (LEfSe) in (a) UC vs HS and (b) PSC-IBD vs HC.

**Supplementary file 1** List of differentially expressed genes and corresponding Log2 fold change (based on FDR corrected P value < 0.05) comparing each of the three cohorts of patients.

**Supplementary file 2** List of Gene Ontology and KEGG/Reactome pathways and the corresponding direction of pathway (based on FDR corrected P value < 0.05) comparing each of the three cohorts of patients.

**Supplementary file 3** List of genes with maximum connections between the three healthy cohorts based on predicting disease modelling.
