# Supplementary material for: A Pilot Integrative Analysis of Colonic Gene Expression, Gut Microbiota, and Immune Infiltration in Primary Sclerosing Cholangitis-Inflammatory Bowel Disease: Association of Disease With Bile Acid Pathways
Source: J Crohns Colitis. 2020 Feb 4;14(7):935–47. doi: 10.1093/ecco-jcc/jjaa021 (PMC7392170; doi:10.1093/ecco-jcc/jjaa021)
Supplement: jjaa021_suppl_Supplementary_Methods [file jjaa021_suppl_supplementary_methods.docx]

**Supplementary Methods**

*RNA library preparation*

Qiagen RNAlater TissueProtect tubes containing mucosal biopsies were thawed on ice. Following mechanical lysis with TissueLyser (Qiagen, Hilden, Germany), the Qiagen AllPrep DNA/RNA Mini Kit was used for extraction of DNA and RNA from biopsies. On column DNAse digestion was done prior to elution of RNA in order to reduce carry-over of contaminating DNA. The RNA was quantified by Qubit (Thermofisher, Massachusetts, US) and quality checked by Tapestation (Agilent, Santa Clara, US). Samples with an RNA integrity number (RIN) of less than 8 were excluded from this study. Ribo-Zero Gold rRNA Removal Kit Epidemiology (Illumina, San Diego, US) was then used to remove contaminating ribosomal RNA and samples were then cleaned up by ethanol / glycogen precipitation. The SMARTer Stranded RNA-Seq Kit (Takara, Kusatsu, Japan) was used for cDNA synthesis and strand-specific library construction. Paired-End 75 bp sequencing was performed using NextSeq 500/550 High Output Kit v2 kits (Illumina, San Diego, US).

*LPMC isolation and staining from colonic biopsies*

A collagenase-DNase digestion mix with 100 units/mL of collagenase and 150 μg/mL DNase was added to the gentleMACS tubes containing mucosal samples in complete RPMI and incubated at 37°C for one hour. Cells in the biopsies were then dissociated further by gentleMACS Dissociator (Miltenyi Biotec, Bergisch Gladbach, Germany) following which the suspension was then passed through a cell strainer, washed with PBS. Lamina propria mononuclear cells (LPMCs) were then isolated by gradient centrifugation using Percoll solution (Sigma Aldrich, St. Louis, US), resuspended in complete RPMI and counted to ensure a minimum yield of 1.5 million.

Cells were distributed for CD4 phenotyping and intracellular cytokine staining panels along with relevant fluorescent minus one controls. For intracellular cytokine staining, cells were incubated with stimulated Cell Activation Cocktail with Brefeldin A (Biolegend, San Diego, US) - a pre-mixed cocktail with optimized concentration of PMA (phorbol 12-myristate-13-acetate), ionomycin, and protein transport inhibitor (Brefeldin A) for 4 hours at 37°C.

The following antibodies were used for flow cytometry staining: Viability dye-APC-Cy7 (eBioscience - Catalogue No :65-0865-14), CD14-APC-Cy7 (Biolegend - Catalogue No :325620), CD19-PE (Biolegend - Catalogue No :302208), CD3-FITC (Biolegend - Catalogue No :344804), CD4-PerCP (Biolegend - Catalogue No :300528), CD127-AF647 (Biolegend - Catalogue No :351317), CD25-PE-Cy7 (Biolegend - Catalogue No :302611), CCR6-BV650 (Biolegend - Catalogue No :353425), CD161-BV711 (BD Biosciences - Catalogue No :563865), CCR5-PE-Texas Red (Biolegend - Catalogue No :359126), CXCR3-BV421 (Biolegend - Catalogue No :353715), FoxP3-APC (eBioscience - Catalogue No :17-4776-41), IL17A-PE (Biolegend - Catalogue No :512306), IFNy-PE-Cy7 (Biolegend - Catalogue No :502528), TNF-APC (Biolegend - Catalogue No :502912).

*Predictive analytics*

We used the Random Forest (RF) machine learning ensemble method to obtain predictive performance of the data. This allowed all the features to be analysed in a nonlinear way rather than solely in a linear way, and hence allowed the discovery of more complex dependencies among features. Random Forest uses a bootstrapping methods for creating a model (called as training set) or for testing the performance of the model. The bootstrapping process generates random samples from the dataset with replacement. Every bootstrapped sample has a corresponding left out or 'out-of-bag' (OOB) sample which is used to test performance of the algorithm. For example, if we generate 1000 bootstrapped samples, each time we will get a set of predictions from the training samples. The final prediction is simply the average of all 1000 predictions from the trees that do not contain training samples in their respective bootstrap sample (test samples). We used RF as a classification method to classify different response, class labels or outcome variables. The class labels or outcome variables were considered as a combinatorial way: PSC-IBD vs. HC; UC vs. HC and PSC-IBD vs. UC patients. For the classification model, RF needed to use some of the parameters to be set a priori. For example, the number of trees (ntree) and the number of variables (for example: number of genes) randomly sampled as candidates at each split (mtry) needed to be defined. We used ntree=500 and mtry =square root of variables in our models. For example, for this data set, mtry value was set to the nearest integer to the square root of the number of features. To select the optimum number of features from each of the data sets, we ranked all the features (each data separately) and gradually took top features and estimated AUC values. This process yielded probable predictive features for further analysis and data integration. Based on the gene interactions we used a cut-off of at least 15 connections/interactions along with genes that were biologically relevant following which they were integrated with significant immunological parameters and 16S microbial profiles. Pearson correlation analysis was performed for data integration.
