# Supplementary material for: A Pilot Integrative Analysis of Colonic Gene Expression, Gut Microbiota, and Immune Infiltration in Primary Sclerosing Cholangitis-Inflammatory Bowel Disease: Association of Disease With Bile Acid Pathways
Source: J Crohns Colitis. 2020 Feb 4;14(7):935–47. doi: 10.1093/ecco-jcc/jjaa021 (PMC7392170; doi:10.1093/ecco-jcc/jjaa021)
Supplement: jjaa021_suppl_Supplementary_Results [file jjaa021_suppl_supplementary_results.docx]

**Supplementary Results**

***Differential gene expression and pathway analysis in pre liver transplant PSC-IBD – subgroup analysis***

Although no differences in gene expression were identified between pre and post liver transplantation PSC-IBD patients, we acknowledge that this is a potential confounder. Consequently we performed a subgroup analysis in pre liver transplant PSC-IBD patients (n=7) of gene expression and pathways in comparison to the UC and HC cohorts. We found 1145 genes were differentially expressed in PSC-IBD (612 upregulated and 533 downregulated) compared to UC and 1194 differentially expressed genes (757 upregulated and 437 downregulated) compared to HC.

Pathway analysis comparing pre-liver transplant PSC-IBD with UC demonstrated significant enrichment of 468 biological processes (106 upregulated and 362 downregulated) and 147 KEGG/Reactomy pathways (47 upregulated and 101 downregulated). Pathways associated with fatty acid metabolic processes, glucuronidation, bile acid and bile salt metabolism processes and transport were upregulated in pre-transplant PSC-IBD compared to UC (Supplementary figure 11). Processes such as those associated with immunological response were downregulated compared to UC.

Pathway analysis comparing pre-liver transplant PSC-IBD with HC revealed enrichment of 468 biological processes in PSC-IBD compared to HC. Of these 363 were upregulated and were primarily associated with innate, adaptive and humoral immune response with upregulation of anti-microbial defence and extracellular matrix remodelling processes (Supplementary figure 12). Similarly, analysis using KEGG/Reactome revealed that 148 pathways that were differentially regulated (101 upregulated) in PSC-IBD compared to HC and associated with various immunological mechanisms.

The key findings of changes in bile acid homoestatic and immunological pathways in pre-transplant PSC-IBD patients were not different in comparison to the PSC-IBD cohort that included three post liver transplant patients with recurrence of PSC possibly suggesting that the colonic mucosal biological processes did not appear to change as a result of liver transplantation. The full list of differentially expressed genes and pathway analysis for this subgroup is provided as supplementary file 4.


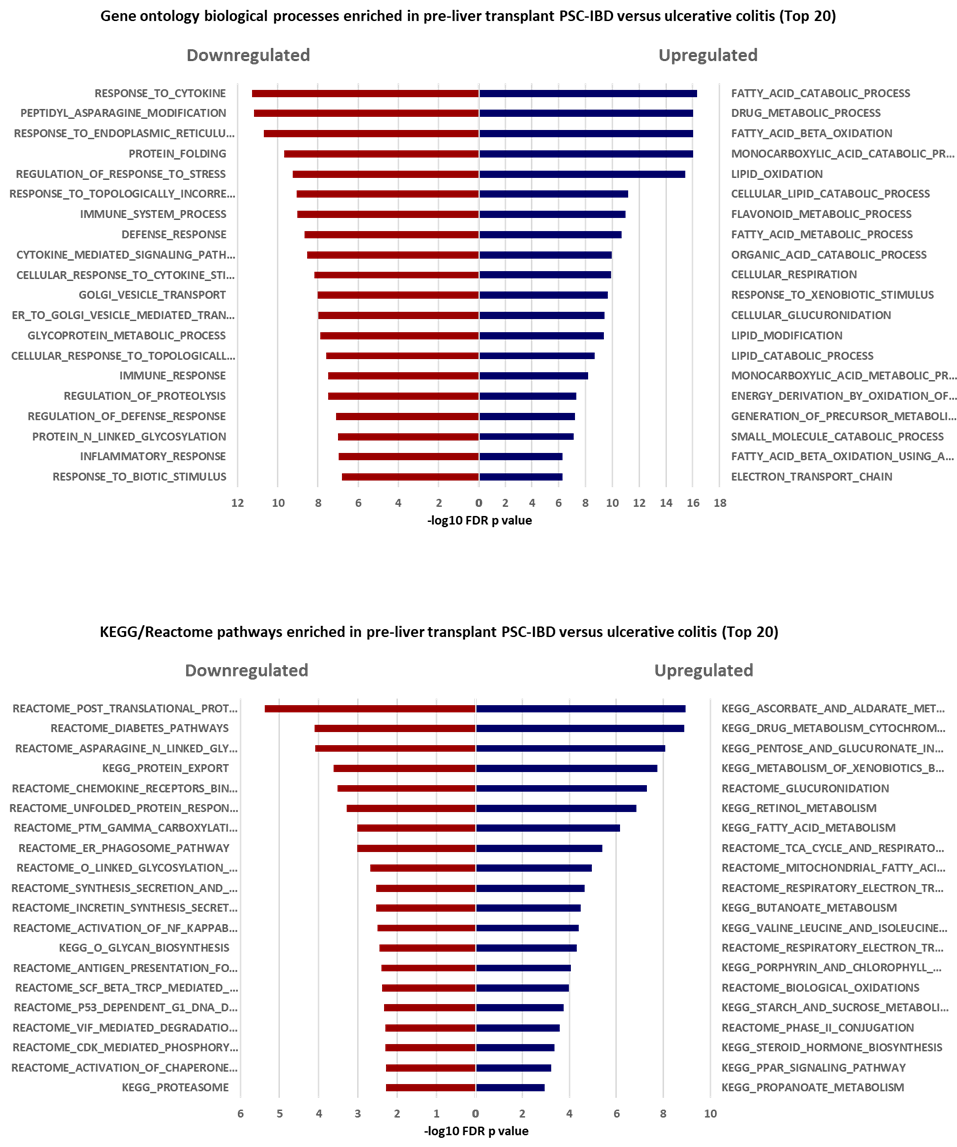


**Supplementary figure 11 – Pathway analysis between pre liver transplant PSC-IBD versus UC**

Top 20 gene ontology biological processes in pre liver transplant PSC-IBD vs UC demonstrate metabolic pathways, many of which are involved in bile acid homeostasis are upregulated in PSC-IBD compared to UC whereas immune activation and defence pathways are upregulated in UC compared to PSC-IBD.


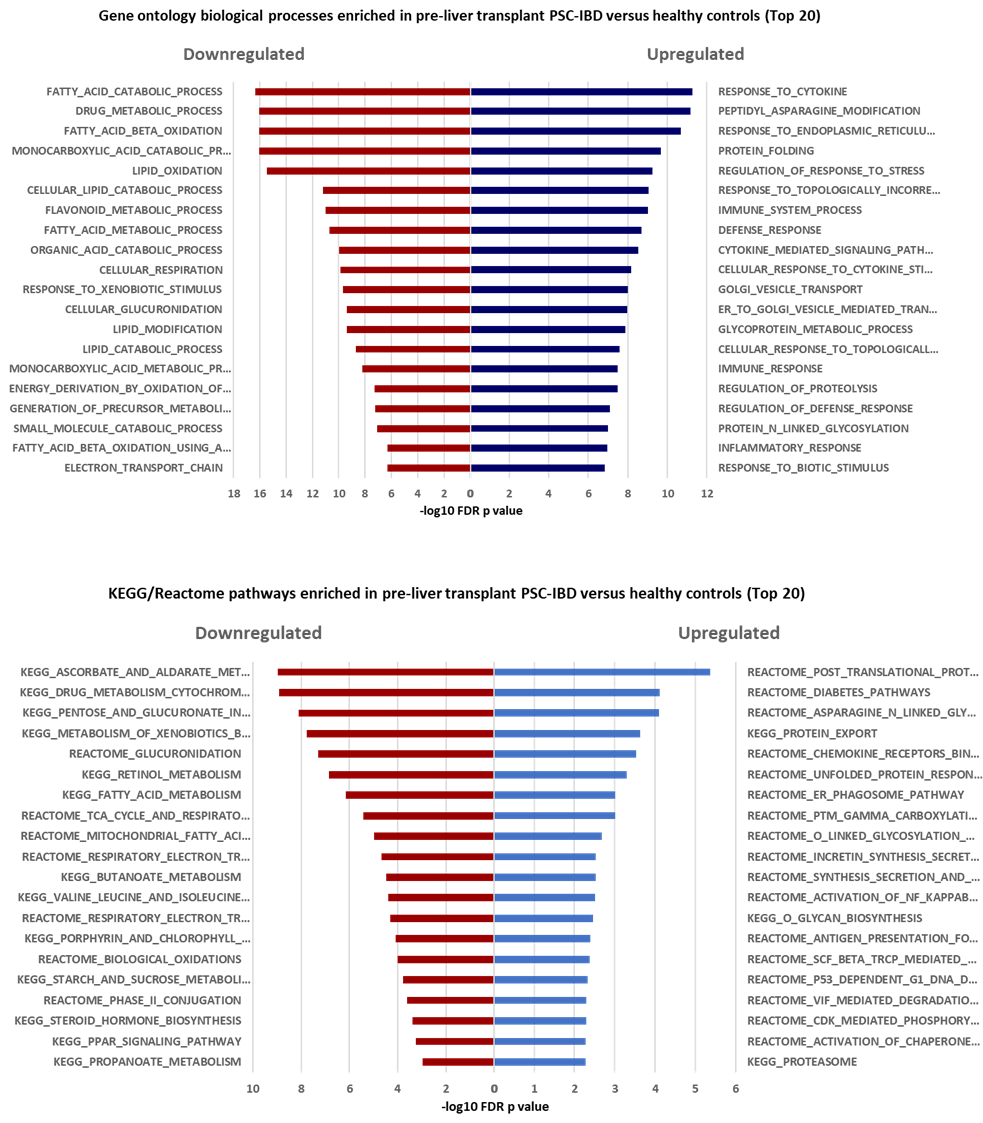


**Supplementary figure 12 – Pathway analysis between pre liver transplant PSC-IBD versus UC**

Top 20 gene ontology biological processes and KEGG/Reactome pathways by competitive gene set testing using Camera demonstrate upregulation of immune mediated pathways in in pre liver transplant PSC-IBD compared to HC.
